# Ecological Momentary Intervention to Replace Sedentary Time With Physical Activity to Improve Executive Function in Midlife and Older Latino Adults: Pilot Randomized Controlled Trial

**DOI:** 10.2196/55079

**Published:** 2024-09-05

**Authors:** Ulf G Bronas, David X Marquez, Cynthia Fritschi, Katherine Petrarca, Spyros Kitsiou, Olu Ajilore, Nathan Tintle

**Affiliations:** 1 School of Nursing Columbia University New York, NY United States; 2 Division of Rehabilitation and Regenerative Medicine Columbia University New York, NY United States; 3 Department of Biomedical and Health Information Sciences University of Illinois Chicago Chicago, IL United States; 4 College of Nursing University of Illinois Chicago Chicago, IL United States; 5 College of Medicine University of Illinois Chicago Chicago, IL United States

**Keywords:** sedentary time, physical activity, cognition, older adults, Latinos, mobile phone

## Abstract

**Background:**

Exercise interventions often improve moderate to vigorous physical activity, but simultaneously increase sedentary time due to a compensatory resting response. A higher level of sedentary time is associated with a lower level of executive function, while increased moderate to vigorous physical activity is associated with improved global cognition and working memory among Latino adults. Latino adults are the fastest-growing minority group in the United States and are at high risk for cognitive decline, spend more time sedentary compared to non-Hispanic populations, and engage in low levels of physical activity. Interventions that are culturally appropriate for Latino adults to replace sedentary time with physical activity are critically needed.

**Objective:**

This study aims to develop and test the feasibility and acceptability of an ecological momentary intervention (EMI; delivered in real time) that is individually designed to replace sedentary time with physical activity in Latino adults.

**Methods:**

This pilot study randomized 39 (n=26, 67% female; mean age 61, SD 5.8 years) community-dwelling, Spanish-speaking Latino adults (1:1 allocation) to either a 6-week EMI program designed to replace sitting time with physical activity (20/39, 51%) or physical activity guidelines education (19/39, 49%). The program was conducted on the web and in Spanish. The intervention was individualized based on individual interview responses. The intervention included the use of a Fitbit activity monitor, weekly didactic phone meetings, interactive tools (SMS text messages), and coach-delivered feedback. Feasibility and acceptability were assessed via study satisfaction (Likert scales), motivation (ecological momentary assessment), retention, and compliance. Sedentary time and physical activity were assessed via 7-day actigraphy. Cognitive performance was assessed via the trail making test part A and B (part B=executive function) and via the National Institutes of Health Toolbox remote cognitive assessment. Statistical analysis included a linear model on change score from baseline, adjusting for age, sex, and education, emphasizing effect size.

**Results:**

Participant satisfaction with EMI was high (9.4/10), with a high degree of motivation to replace sitting time with physical activity (9.8/10). The intervention compliance rate was 79% with low difficulty using the Fitbit (1.7/10). Weekly step count increased in the intervention group by 5543 steps (group difference: *d*=0.54; *P*=.05) and sedentary time decreased by a mean 348 (SD 485) minutes (group difference: *d*=0.47; *P*=.24) compared to controls, with moderately strong effect sizes. The trail making test part B improved in the intervention group (mean –35.26, SD 60.35 seconds), compared to the control group (mean 7.19, SD 46 seconds; group difference: *d*=0.74; *P*=.01). No group differences were observed in other cognitive measures.

**Conclusions:**

An individualized EMI designed for midlife and older Latino adults has the potential to replace sitting time with physical activity and improve executive functioning. The intervention was feasible and well received with a high degree of satisfaction.

**Trial Registration:**

ClinicalTrials.gov NCT04507464; https://tinyurl.com/44c4thk5

## Introduction

### Background

The US population of adults aged ≥65 years is projected to increase from 58 million in 2021 to approximately 88 million by the year 2050 [1]. Concurrently, it is estimated that 13.8 million Americans will be diagnosed with Alzheimer disease and related dementias (ADRD) by that same year [2]. This is a significant increase from the approximately 5 million diagnosed with ADRD in 2014 [3]. The Latino/a/x/e (hereafter referred to as Latino) population within this age cohort will comprise 18% of the total US population by 2050 [4]. The growth projected within this subgroup has significant implications for the burden of ADRD, as it has been well established that rates of ADRD are disproportionately higher among older Latino adults compared to other ethnicities of the same age [1,4-6]. Currently, >12% of older Latino adults have been diagnosed with ADRD [6]. Even more striking, the number of Latino adults with ADRD is expected to increase 7-fold over the next 3 decades, representing an epidemic increase of ADRD [3]. The high incidence of ADRD in conjunction with physical function limitations places older Latino adults at a high risk for loss of independence and significant caregiver burden.

In addition, older Latino adults have a higher prevalence of risk factors for cognitive decline, and the rate of increase in risk factors is greatest among Latinos [7,8]. Latinos further engage in less leisure time physical activity and spend more time sedentary, compared to non-Latino White participants, ranging from 46.8% to 74.2% versus 34.3% to 58.2% [9,10]. Thus, increasing physical activity is of tremendous significance to this population. Of note, physical activity programs that have successfully increased daily physical activity in older Latino adults often report an increase in sedentary time [11-15]. Sedentary time, defined as not raising energy expenditure above 1.5 metabolic equivalents and includes lying down, sitting, watching television, and other screen-based entertainment activities, is a known risk factor for reduced cognitive function, especially executive function, in older Latino adults [16-19]. Thus, studies are clearly needed to investigate the benefits of breaking up and replacing sedentary time with physical activity on cognition and brain connectivity in older Latino adults [20-22]. However, to develop and deliver a successful intervention, we first need to understand how to best conduct a lifestyle intervention to replace sitting time with physical activity in the Latino community following stage 1A to 1B in the National Institutes of Health (NIH) model and assess feasibility and acceptability.

The current view of the field is rooted in a Eurocentric view, which assumes that interventions that work in one community will work the same in a different community. This assumption is likely incorrect and represents a significant gap in our ability to develop a successful intervention at the multiple levels that are required across the socioecological model (SEM) to be successful [23,24]. Thus, developing and testing the feasibility and acceptability of an individually, culturally appropriate, and community-based ecological momentary intervention (EMI; delivered in real time during participants’ everyday lives) on replacing sedentary time with physical activity and cognitive performance is warranted. In addition, including participants in that latter part of midlife is tremendously important because this represents a window of opportunity for behavior change to allow for healthy aging. Due to the heterogeneity and intricacies of midlife and older Latino adults in the Chicago area and to ensure cultural validity of the EMI, we need to understand factors that facilitate initiation of and adherence to behavioral change at the individual level using the social cognitive theory [25-27].

### This Study

Following the NIH model stage 1A to 1B, this study aimed to determine the feasibility and acceptability of a 6-week EMI program and explore effect sizes in the change in sedentary time, daily physical activity, and cognitive performance in midlife and older Latino adults, delivered in the home-setting over 6 weeks, compared to participants randomized to receive physical activity guidelines in Spanish. We hypothesized that the EMI program would be feasible and acceptable and that effect sizes would indicate plausibility of (1) reduced sedentary time (secondary outcome), (2) increased device-assessed daily physical activity (secondary outcome), and (3) improved cognitive performance (executive functioning and cognitive flexibility, and secondary outcome), compared to a group randomized to receive education on physical activity guidelines.

## Methods

### Overview

This study was a pilot of a 2-armed, parallel group randomized controlled trial. All procedures were conducted during COVID-19 restrictions between November 2020, and February 2022, remotely via telephone and videoconference. The principal investigator and assessor were blinded to group assignment. Research staff enrolled participants. Participants were randomized by the statistician using computerized variable block randomization of 2 and 4 as designed by the project statistician not involved in recruitment. All outcome assessments were conducted 1 week before randomization and 1 week following completion of the study period. Allocation was 1:1. The study was registered with ClinicalTrials.gov (NCT04507464) and reported in accordance with the CONSORT (Consolidated Standards of Reporting Trials) 2010 statement: extension to randomized pilot and feasibility trials checklist [28].

### Sample Size Consideration

A sample size of 12 per group is recommended for pilot studies estimating the mean and variability of continuous outcomes [29]. We, therefore, aimed to enroll 40 participants in this pilot study to ensure completion with an adequate sample size accounting for a possible 10% to 20% attrition.

### Recruitment

We recruited participants through flyers posted at local Latino-based organizations and on websites, as well as using word of mouth and through media advertisements. Investigators also gave Zoom presentations at several primarily Spanish-speaking churches to recruit additional participants in the Chicago area. We assessed 69 participants for eligibility.

Inclusion criteria were (1) being aged 55-89 years, (2) having no major head trauma, and (3) owning a working smartphone with internet and a messaging plan. Exclusion criteria were participating in a supervised exercise program, being wheelchair dependent, self-reporting a diagnosis of dementia, using medications to improve cognition or mood, and having contraindications to exercise per American College of Sports Medicine guidelines [27].

### Measures

#### Timing of Measures

Participants were first screened to determine eligibility via telephone, and those who met the criteria completed the informed e-consent (in REDCap [Research Electronic Data Capture]; Vanderbilt University) conducted via Zoom (Zoom Video Communications, Inc) video. Once consent was obtained, participants provided basic demographic information (age, sex, marital status, ethnicity, preferred language, and education) and a full medical history. We then completed the Charlson comorbidity questionnaire for each participant and conducted the following NIH Toolbox cognitive function measures as approved by the NIH for use via video call [30]: picture vocabulary test, picture sequence test, list sorting test, oral reading recognition test, auditory verbal learning test, and the auditory trail making test part A (TMT-A) and trail making test part B (TMT-B) [31-34]. TMT-B time to completion was considered the measure of executive function.

Following cognitive function testing, we conducted a video interview using Zoom or Teams (Microsoft Corp), which was recorded, transcribed, and translated for the development of an individualized, culturally tailored EMI program (see the Intervention section for details). Once participants completed the interviews, they were mailed a triaxial accelerometer (ActiGraph GT3X+, ActiGraph), which they were instructed to wear on their right hip and waist for 7 consecutive days to measure sedentary time and daily physical activity. Upon return of the ActiGraph, participants were randomized and notified of their assigned group (EMI or physical activity guideline education). The accelerometer was worn by both groups at baseline and at follow-up. Data collectors instructed the participants on the use of the accelerometer and the use of an accelerometer log.

#### Primary Outcome Measures

The primary outcome measures were feasibility and acceptability. Study feasibility and acceptability measures were assessed by study satisfaction debriefing at the end of the study via 10-point Likert scales. Satisfaction Likert scales were as follows: (1) How satisfied were you with the program? (0=not satisfied-10=extremely satisfied), (2) Did Fitbit motivate you to exercise? (0=not helpful-10=extremely helpful), (3) Did you enjoy the physical activity options provided by SMS text message? (0=not at all-10=very much), and (4) Did you have difficulty using Fitbit and the app? (0=no difficultly-10=very much difficulty). We further asked an open-ended question, Is there anything you would like us to know about the program that is good or that we should improve? (sample quotes are provided in Table 1). Acceptability of the program and instruments was defined as 70% (14/20) of participants rating >8/10 on study satisfaction and <2/10 on difficulties the Likert scale measures based on consensus and previous research conducted in the Bronas laboratory.

Intervention delivery, feasibility, and acceptability were measured via ecological momentary assessment (EMA) during the course of the intervention period using 10-point Likert scales. EMA assessment provided real-time feedback 3 times per day during the study following SMS text message prompts using several Likert scales: (1) Did the SMS text message motivate you to exercise? (0=not at all-10=very much), (2) Did you enjoy the SMS text message prompt? (0=not at all-10=very much), (3) Did you have difficulty with understanding the SMS text message? (0=no difficulty-10=very much difficulty), and (4) Did the SMS text message help you with the Fitbit? (0=not at all-10=very much). Acceptability and feasibility of the intervention delivery were defined as 70% of participants rating >8/10 on study satisfaction and <2/10 on difficulties in the EMA Likert scale measures based on consensus and previous research in the Bronas laboratory.

Feasibility was further defined as a participant retention rate of >80%, a compliance rate to the intervention of >70% (defined as <70% of time spent sedentary or at least 150 min of moderate physical activity per week), and a wear time of activity tracking devices >80%. Compliance was assessed using the Fitbit to assess the fidelity of treatment. All outcome measures were obtained at baseline 1 week before randomization and 1 week after completion of the study period. To analyze data obtained from the individual phone interviews, we conducted a thematic analysis to explore themes. The thematic analysis will provide a deeper understanding of physical, psychological, and social influences that influence physical activity choices in midlife and older Latino adults. Understanding these factors will be crucial for the continued refinement of effective interventions.

**Table 1:** Baseline demographics.

Variables		Full sample (N=39)	Intervention (n=20)	Control (n=19)	*P* value^a^
**Demographics**					
	Age (y), mean (SD)	61 (5.85)	59.9 (4.99)	62.16 (6.57)	.24
	Sex (female), n (%)	26 (67)	11 (55)	15 (79)	.17
	Married, n (%)	32 (82)	19 (95)	13 (68)	*.04^b^*
	Employed, n (%)	20 (51)	12 (60)	8 (42)	.35
	Years education, mean (SD)	12.21 (4.53)	13.35 (4.59)	11 (4.24)	.11
	BMI (kg/m^2^), mean (SD)	29.7 (4.91)	29.97 (4.05)	29.41 (5.78)	.73
	Previous exerciser^c^, n (%)	18 (46)	10 (50)	8 (42)	.75
**Medications, n (%)**					
	Blood pressure	11 (28)	6 (30)	5 (26)	.99
	Calcium channel blocker	4 (10)	2 (10)	2 (11)	.99
	β-Blocker	2 (5))	1 (5)	1 (5)	.99
	Diuretics	1 (3)	0 (0)	1 (5)	.48
	Blood thinners	3 (5)	1 (5)	1 (5)	.99
	ACE^d^ or ARB^e^	7 (18)	3 (15)	4 (21)	.70
	Diabetes	8 (21)	6 (30)	2 (11)	.23
**Medical history, n (** **%)**					
	CAD^f^	1 (3)	0 (0)	1 (5)	.48
	Hypertension	11 (28)	5 (25)	6 (32)	.74
	Diabetes	6 (15)	5 (25)	1 (5)	.19
	Lung disease	7 (18)	3 (15)	4 (21)	.69

^a^*P* values from 2-sample *t* tests or chi-square tests, using a Monte Carlo simulated *P* value (10,000 replicates) to account for the small sample sizes and cell counts.

^b^Italicized values indicate *P*<.05.

^c^Meeting surgeon-general recommendation.

^d^ACE: angiotensin-converting enzyme.

^e^ARB: angiotensin receptor blocker.

^f^CAD: coronary artery disease.

#### Secondary Outcome Measures

The secondary outcome measures included sedentary time and cognitive function. Sedentary time, measured using the ActiGraph, was defined as an awake activity with an energy expenditure ≤1.5 metabolic equivalents occurring in a sitting or reclining posture, vector magnitude counts of <70 counts/15 seconds, and vertical axis counts of <10 counts/15 seconds. The sedentary interruption was defined as counts >100/minute. Nonwear time was considered as 60 minutes of 0-activity counts. Any counts >15,000 were considered erroneous [35]. A valid day consisted of 10 waking hours and at least 3 complete days of data (1 person) had to be available to be included in the final analysis [36]. Data were processed with ActiLife (version 6.13.5) software, with data converted to 60-second epochs. Nonwear time was defined as at least 60 consecutive minutes of 0 activity count. We categorized physical activity according to the study by Freedson et al [37] cutoff points. Cognitive function was assessed using the auditory TMT-A and TMT-B, and the NIH Toolbox measures remote assessment [30]. We captured Fitbit data remotely in real time for compliance and fidelity of treatment to the EMI program using a digital health platform (iCardia; Spyros Kitsiou; University of Illinois, Chicago) [38].

### Intervention

The EMI program was developed based on the principles of the SEM, by understanding and considering participants’ individual cultural interaction with their living environment [39]. We individualized and adjusted the program based on participants’ stage of behavior change following the transtheoretical model using SMS text messages designed in part with participant input that included health outcomes (eg, goal setting, self-monitoring, problem-solving barriers, and increasing social support). The EMI program was also designed to provide motivational feedback of successes and encouragement via individualized SMS text messages (eg, specific goal achievement) to empower participants in their own ability to achieve behavior change, gain positive results, and improve self-efficacy beliefs as explained by the social cognitive theory [40]. We achieved this by interviewing each participant. Participants told investigators what times during the day they wanted the SMS text message reminders and how the SMS text messages should be phrased and what they preferred to do for physical activity based on their living environment. They also provided their preference for which health benefit reminders to be sent, and they helped design these to match each individual preference. Participants further told investigators what type of feedback they preferred and when they preferred the delivery of SMS text message feedback of successes. This was conducted for all participants during the initial interview before randomization. SMS text messages were adjusted based on participant feedback. This was only in the context of sedentary behavior. Although goal achievement feedback was individualized, all EMI participants received a weekly SMS text message summarizing completed sedentary time, steps, and physical activity and encouragement of successes.

Participants in the EMI group were mailed a Fitbit Charge 4 activity monitor, and a research assistant helped them to set it up via Zoom and configure it to receive sedentary behavior notifications in the form of vibrations when a participant walked <250 steps per hour during waking hours (lowest setting available for the Fitbit; waking hours were selected by the participant). We then scheduled a video call to set up a Health Insurance Portability and Accountability Act–compliant EMA system (Ilumivu mEMA [Ilumivu Inc]) and provided education about Fitbit and the intervention to disrupt sedentary time. Through the use of the Fitbit activity tracker and SMS text messages, we were able to implement real-time delivery of the behavior options, and the Ilumivu mEMA [41] smartphone app allowed us to obtain feedback on the effectiveness of their behavior choices. Although the intervention was guided by the individual interview findings, in general, participants received suggestions on their smartphones on how to replace sitting time with physical activity, such as standing up 5 times, taking 20 steps, or even performing a short (20 s) preferred dance routine. Participants received 3 SMS text messages daily sent from the iCardia platform via the SMS text message service platform Twilio (Twilio Inc). They further received reminders on their smartphones to enter real-time feedback on activity options selected and how successful they were in adopting the option. These data allowed us to track underlying preferences for behaviors and tailor the program accordingly to each individual.

Participants received a message about the success of positive choices for behavioral action. The resulting actions and delivery success of the intervention were automatically captured and downloaded in real time using iCardia and the Ilumivu mEMA smartphone app. Participants randomized to the control group were sent and received an hour-long video education on general physical activity guidelines (questions were entertained and answered). The control group was called weekly to keep them engaged in the study, but due to the limited nature of the pilot study, they did not receive a Fitbit, and thus, this was not a true attention control group. After the completion of the 6-week intervention period, all participants were again mailed an ActiGraph to be worn on the waist for 7 days to measure sedentary time and daily physical activity. Once the ActiGraph was mailed back, a phone interview was scheduled with each participant to understand where they had difficulty and what aspects of the program did or did not work for them. The cognitive function tests administered at baseline were then repeated.

### Statistical Analysis

This was a pilot randomized controlled trial for feasibility, acceptability, and to determine preliminary effect sizes. Therefore, we emphasized descriptive statistics, such as means, SDs, frequencies, percentages, and effect sizes, to demonstrate the feasibility of recruitment, compliance, retention, treatment effects over time, and proof of concept. In addition, 2-sample *t* tests and chi-square tests (using Monte Carlo simulation to obtain *P* values) were used to test for baseline demographic differences across randomized groups. When evaluating primary and secondary outcomes, paired *t* tests were used to assess within-group change from baseline to 6 weeks. Cohen *d* was used to estimate the effect of the intervention (change in EMI vs control). Linear models predicting change scores by treatment group (unadjusted model), and similar models adjusting for age, sex, and years of education were computed for each outcome measure. *P*<.05 with 2-sided tests was used to assess statistical significance, though *P*<.20 was considered a trend toward significance given the pilot nature of the study.

### Ethical Considerations

This study was approved by the University of Illinois at Chicago Institutional Review Board (2020-0739), and informed consent was obtained before any study procedures.

## Results

### Overview

**Figure 1:**
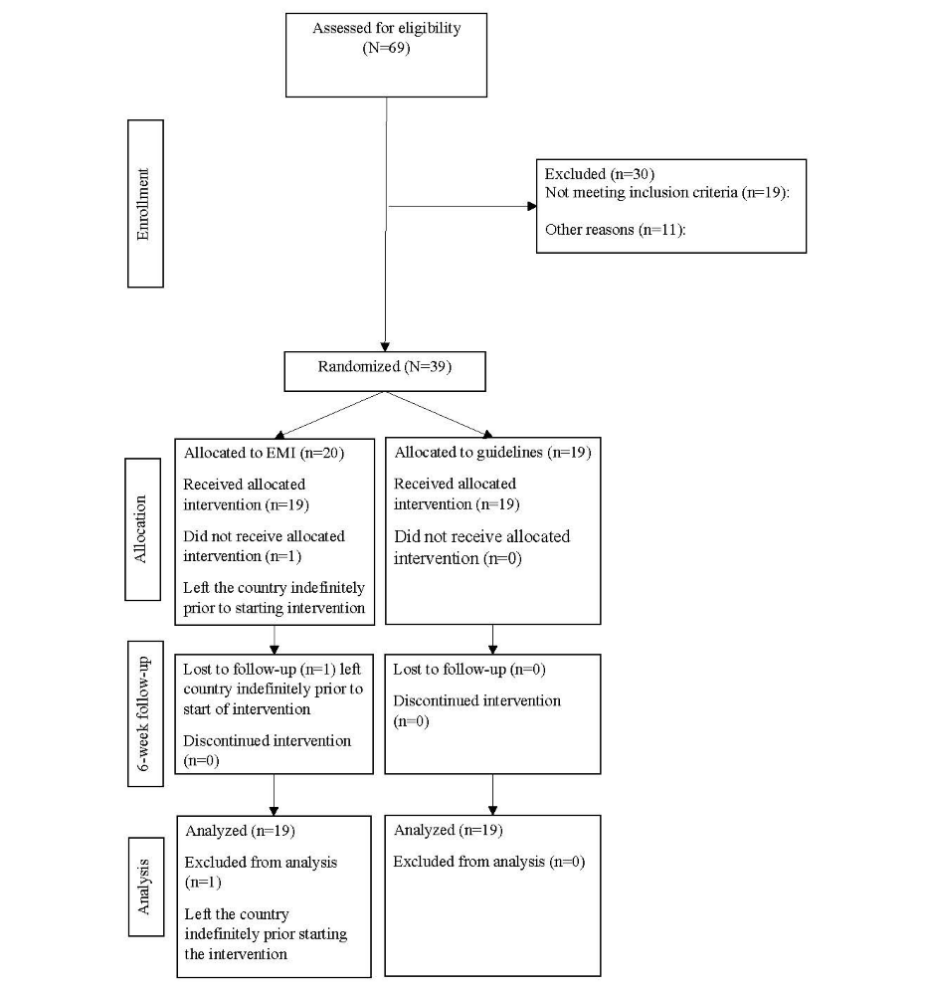
CONSORT (Consolidated Standards of Reporting Trials) diagram. EMI: ecological momentary intervention.

Of the 69 participants assessed for eligibility, 19 (28%) did not meet the inclusion criteria, and 50 (72%) participants met the inclusion criteria. Of the 50 participants that met the inclusion criteria, 11 (22%) participants declined participation due to disinterest and time constraints. Therefore, 39 (78%) participants were randomized to either the EMI (n=20) or the group receiving education on physical activity guidelines (control; n=19). Moreover, 1 (3%) participant was lost to follow-up after randomization, but before intervention initiation due to leaving the country permanently (CONSORT flow diagram; Figure 1) and was not included in analyses because the intervention did not begin, and we were interested primarily in the feasibility and acceptability of the intervention.

The 2 randomized groups did not show evidence of difference in baseline demographics, medication use, and medical history, except for the EMI group consisting of slightly more individuals indicating they were married (Table 1). Most (37/39, 95%) of the samples identified as Latino only with only 2 (5%) of the 39 participants identifying as Hispanic or Latino White. The sample was representative of the Chicago Latino community in terms of education albeit slightly below those published in the Chicago 2022 Community Survey [42]. When compared with the Chicago 2022 Latino Community Survey [42], 32% versus 29% had lower than a high school education, 34% versus 28% had a high school education, and 15.8% versus 20% had an associate’s or bachelor’s degree.

### Primary Outcome: Feasibility and Acceptability

We were able to recruit a representative sample virtually during COVID-19 restrictions. Of the 69 participants accessed for eligibility, 50 (72%) met the inclusion criteria. Of the eligible 50 participants 39 (78%) were enrolled in the study indicating the feasibility of recruitment. Although we aimed to enroll 40 participants, we elected to stop enrollment at 39 (98%) participants. This was due to the COVID-19 restrictions limiting further outreach efforts and meeting criteria of 12 per group for pilot studies. The retention rate was high with only 1 (3%) loss to follow-up and no missing data on outcome measures. Feasibility and acceptability of EMI included the 19 (95%) of the 20 participants who initiated the EMI program. The participants expressed a high degree of acceptability, and study satisfaction was high (Table 2). Compliance with the EMI program was 79% defined as <70% awake time spent sedentary or 150 minutes of moderate activity per week, and the mean minutes of activity minutes per week were 382 minutes per week captured via Fitbit. There were no adverse events associated with study procedures in either group. A total of 2850 SMS text messages were sent out. All of them were successfully delivered (100% rate). EMA survey results from 1394 survey messages over 6 weeks showed that EMI participants found the SMS text message suggestions to move to be highly motivating 82.4% of the time (8-10 on a 0-10 Likert scale). EMI participants enjoyed the text messages 84.4% (8.44/10, on a 0-10 Likert scale), and participants found that the Fitbit was motivating in conjunction with receiving SMS text message suggestions to move 90.9% of the time (9.09/10 on a Likert scale), while 92.7% found no little-to-no difficulty using the Fitbit tracker and mobile app (0-2 on a 0-10 Likert scale). Participants reported feeling highly motivated by the SMS text messages encouraging them to break up sedentary time and partake in alternative forms of exercise. When asked about the effectiveness of the program in motivating them to exercise, 1 participant reported, “This program motivated me so much because it helped me do more exercise and movement. It helped me open my mind to more exercises I can do to stay motivated.” Many participants provided similar feedback, such as the following quote: “the Fitbit motivates me to do the exercises at the time I receive the messages and the program works well to motivate the people to do exercise. I believe people need that sort of motivation.”

**Table 2:** Study satisfaction participants randomized to the ecologic momentary intervention^a^.

Program aspect (n=19)	Participants’ perception (0-10)	Sample quote
Program satisfaction	9.4 (range: 8-10)	“I would like more people to participate in the program because it motivates people to be in movement. This program helped me to do more exercise and be in movement than before; it reminds me to be active. This program motivated me so much because it helped me do more exercise and movement. It helped me open my mind to more exercises I can do to stay motivated.”
Fitbit motivation	9.8 (range: 9-10)	“Yes, it helps us be involved more in exercises and be active at home too. With this program, you are reminded to be active, such as giving us the Fitbit watch. I loved the Fitbit watch and the motivation it gave me, especially telling me the number of steps, calories I burned throughout the day. I highly recommend this program to everyone; it helped me a lot.”
SMS text message enjoyment	9.5 (range: 8-10)	“The Fitbit motivates me to do the exercises at the time I receive the messages, and the program works well to motivate the people to do exercise. I believe people need that sort of motivation.”
Difficulty using Fitbit	1.7 (range: 0-10)^b^	“I have participated in many studies before, and this exercise study has motivated me to do more steps every day with the use of the Fitbit.”

^a^Program satisfaction (0=not satisfied-10=extremely satisfied). Motivation (0=not helpful-10=extremely helpful). Enjoy SMS text message (0=not at all-10=very much). Difficulty using the Fitbit (0=no difficulty-10=very much difficulty).

^b^One participant had significant difficulties with the Fitbit while switching mobile phones.

### Secondary Outcome: Device-Assessed Sedentary Time

A variety of measures of sedentary time were obtained from ActiGraph data (Table 3). Across all 12 measures, the EMI group showed greater improvement in sedentary time as compared to the control group (fewer sedentary bouts, more sedentary breaks, and less sedentary time overall). A total of 5 (42%) of the 12 measures showed statistically significant improvement (*P*<.05) in the EMI group from baseline, with an additional 4 (33%) measures showing a positive trend (*P*<.12). Evidence of change was much less in the control group (*P*>.50 for 9, 75% of 12 measures, and *P*>.13 in all cases). Overall effect size estimates (Cohen *d*) comparing the 2 treatments were >0.3 for 9 (75%) of 12 measures and >0.05 in all 12 (100%) cases; however, these unadjusted (and adjusted) effects did not attain statistical significance (*P*>.10 in all cases).

**Table 3:** Measures of sedentary time.

Variables			Intervention (n=19)				Control (n=19)					Unadjusted Cohen *d*			*P* value for difference in effect between groups		
			Change^a^, mean (SD)		Paired *t* test (2-tailed; *df*)		Change^a^, mean (SD)		Paired *t* test (2-tailed; *df*)					Unadjusted			Adjusted^b^
**Sedentary bouts**																	
	Total number of sedentary bouts	–11.58 (22.02)		*0.03 (18)^c^*		–2.47 (28.06)		0.71 (19)		0.36			.27			.19	
	Total time spent in sedentary bouts (min)	–347.7 (484.98)		*0.006 (18)^c^*		–27.53 (811.07)		0.88 (19)		0.47			.15			.16	
	Average length of sedentary bout (min)	–1.87 (4.21)		0.07 (18)		0.09 (3.2)		0.9 (19)		0.51			.12			.34	
	Minimum length of sedentary bouts (min)	0.01 (0.11)		0.83 (18)		–0.01 (0.17)		0.79 (19)		0.14			.73			.70	
	Maximum length of sedentary bouts (min)	–30.26 (129.39)		0.32 (18)		11.75 (83.27)		0.55 (19)		0.38			.24			.38	
	Daily average of sedentary bouts (min)	–40.41 (54.59)		*0.005 (18)^c^*		–9.26 (95.55)		0.68 (19)		0.4			.23			.29	
	Sedentary time (min)	–297.52 (774.12)		0.11 (18)		–31.94 (950.56)		0.89 (19)		0.31			.35			.35	
**Sedentary breaks**																	
	Total time spent in sedentary breaks	731.43 (2423.6)		0.20 (18)		595.62 (1928.07)		0.19 (19)		0.06			.85			.80	
	Mean daily length of sedentary breaks	35.02 (57.34)		*0.02 (18)^c^*		15.6 (61.59)		0.28 (19)		0.33			.32			.39	
	Mean daily time of sedentary breaks	187.43 (425.24)		0.07 (18)		108.33 (293.6)		0.13 (19)		0.22			.51			.81	
	Percent of time sedentary per day	–2.56 (6.25)		0.09 (18)		0.01 (6.55)		0.99 (19)		0.4			.22			.19	

^a^Computed as 6 weeks minus baseline.

^b^Linear models on change scores adjusted for age, sex, and years of education.

^c^Italicization indicates statistical significance (*P*<.05).

### Secondary Outcome: Device-Assessed Daily Physical Activity

A variety of measures of daily physical activity were obtained from ActiGraph data (Table 4). Across all 11 measures, the EMI group showed greater improvement in activity as compared to the control group (light, moderate, and vigorous activity min/d as well as percent of daily activities). A total of 3 (27%) of the 11 measures showed statistically significant improvement (*P*<.05) in the EMI group from baseline, with an additional 3 (27%) measures showing a positive trend (*P*<.15). Evidence of change was much less in the control group (*P*≥.40 for all 11 measures). Overall effect size estimates (Cohen *d*) comparing the 2 treatments were ≥0.3 for 6 (55%) of 11 measures (with 4, 36% of the remaining measures having *d*>0); however, unadjusted (and adjusted) effects did not attain statistical significance in most cases (*P*<.05).

**Table 4:** Measures of physical activity.

Variables			Intervention (n=19)					Control (n=19)					Unadjusted Cohen *d*			*P* value for difference in effect between groups		
			Change^a^, mean (SD)		Paired *t* test (2-tailed; *df*)		Change^a^, mean (SD)			Paired *t* test (2-tailed; *df*)					Unadjusted			Adjusted^b^
**Minutes of activity**																		
	Low-light (min)	19.09 (170.73)		0.63 (18)		4.9 (234)			0.93 (19)		0.07			.83			.65	
	Highlight (min)	8.5 (162.13)		0.82 (18)		–23.41 (185.95)			0.59 (19)		0.18			.58			.43	
	Low-moderate (min)	–12.6 (183.58)		0.77 (18)		–12.69 (138.62)			0.69 (19)		0			.99			.56	
	High-moderate (min)^c^	38.89 (109.85)		0.14 (18)		–18.05 (91.93)			0.4 (19)		0.55			.09			*.03* ^d^	
	Vigorous (min)	25.26 (57.07)		0.07 (18)		–0.44 (24.29)			0.94 (19)		0.57			.08			.30	
	MVPA^e^ (min)	51.56 (282.37)		0.44 (18)		–31.18 (228.03)			0.56 (19)		0.32			.33			.18	
Step count			5542.79 (18,936.52)		0.22 (18)		–3748.58 (13,909.13)			0.26 (19)		0.54			.09			.05
Steps/min			1.16 (2.87)		0.09 (18)		–0.46 (2.29)			0.4 (19)		0.6			.06			*.05* ^d^
**Daily activity percentages**																		
	Low-light	0.89 (1.56)		*0.02* *(18)*		0.16 (2.59)			0.78 (19)		0.34			.30			.31	
	Highlight	0.38 (2.26)		0.47 (18)		–0.09 (2.3)			0.87 (19)		0.21			.53			.57	
	Low-moderate	0.04 (2.51)		0.95 (18)		0.12 (2)			0.8 (19)		0.04			.91			.72	
	High-moderate	0.82 (1.56)		*0.03* *(18)*		–0.22 (1.41)			0.51 (19)		0.67			*.03* ^d^			*.02* ^d^	
	Vigorous	0.43 (0.84)		*0.04* *(18)*		0.01 (0.41)			0.92 (19)		0.61			.06			.29	

^a^Computed as 6 weeks minus baseline within groups.

^b^Linear models on change scores adjusted for age, sex, and years of education.

^c^High to moderate intensity is considered to be 5 to 6 metabolic equivalents.

^d^Italicization indicates statistical significance (*P*<.05).

^e^MVPA: moderate to vigorous physical activity.

### Secondary Outcome: Cognitive Performance

Cognitive performance was assessed with the TMT-A and TMT-B as well as the NIH Toolbox, with 9 total assessed measures (Table 5). Across all 11 measures, the EMI group showed greater improvement in cognitive performance as compared to the control group. In the EMI group from baseline, 1 (11%) of the 9 measures showed statistically significant improvement (*P*<.05), with an additional 4 (44%) measures showing a positive trend (*P*<.20). Evidence of change was much less in the control group (*P*≥.40 for 8/9, 89% of measures). Overall, effect size estimates (Cohen *d*) comparing the 2 treatments were ≥0.3 for 5 (56%) of 9 measures (with the 4, 44% remaining measures having *d*>0); however, unadjusted (and adjusted) effects did not attain statistical significance in most cases (*P*<.05).

**Table 5:** Measures of cognitive performance.

Variables			Intervention (n=19)				Control (n=19)				Unadjusted Cohen *d*		*P* value for difference in effect between groups		
			Change^a^, mean (SD)		Paired *t* test (2-tailed; *df*)		Change^a^, mean (SD)		Paired *t* test (2-tailed; *df*)				Unadjusted		Adjusted^b^
**Trail making measures**															
	Trail making test part A (s)	0.43 (1.27)		0.16 (18)		0.31 (1.73)		0.45 (19)		0.08		.81		.50	
	Trail making test part B (s)	–35.26 (60.35)		*0.02* *(18)* ^c^		7.19 (46)		0.5 (19)		0.74		*.02* ^c^		*.006* ^c^	
	Trail making test part B (errors)	–1.05 (2.8)		0.12 (18)		0.37 (2.24)		0.48 (19)		0.55		.09		*.04* ^c^	
**NIH^d^ Toolbox measures**															
	Picture vocabulary	0.74 (6.52)		0.63 (18)		1 (5.47)		0.44 (19)		0.04		.89		.90	
	List sorting	1.26 (8.7)		0.53 (18)		5.53 (9.64)		*0.022* *(19)* ^*c*^		0.46		.16		.31	
	Picture sequence	4.26 (13.22)		0.18 (18)		4.32 (10.73)		0.097 (19)		0.01		.99		.79	
	Oral reading recognition	6.89 (19.14)		0.13 (18)		1 (7.58)		0.57 (19)		0.4		.22		.27	
	Crystallized cognition	3.95 (10.46)		0.12 (18)		0.95 (5.26)		0.44 (19)		0.36		.27		.27	
	Auditory verbal	0.26 (4.04)		0.78 (18)		0.47 (5.23)		0.7 (19)		0.05		.89		.4	

^a^Computed as 6 weeks minus baseline.

^b^Linear models on change scores adjusted for age, sex, and years of education.

^c^Italicization indicates statistical significance (*P*<.05).

^d^NIH: National Institutes of Health.

### Exploratory Mediation Analysis

On the basis of previous data from Dr Bronas laboratory and the study by Erickson et al [43], we prespecified to explore the potential mediation of moderate to vigorous physical activity (MVPA) on executive function (measured by TMT-B). In unadjusted models, the association of intervention and change in TMT-B was 42.46 seconds (*P*=.02), and the association of intervention and change in percent MVPA was 1.04 (*P*=.04). The association of change in TMT-B and change in percent MVPA was –17.47, indicating that 1% increase in exercise was associated with a 17.47-second improvement in TMT-B (*P*=.003).

After accounting for the change in exercise, the association of intervention and change in TMT-B was 27.42 seconds (*P*=.12). This exploratory analysis provides promising results and suggests that approximately 35% of the impact of the intervention on TMT-B could be accounted for by changes in percent MVPA.

## Discussion

### Principal Findings

This study investigated the feasibility and acceptability of an individualized cultural EMI program for breaking up and replacing sedentary time (sitting) with physical activity on cognitive performance in older Latino adults with Spanish-speaking preference. We were able to recruit a representative sample using virtual recruitment strategies indicating that our recruitment methods were successful and that future findings will be generalizable. A high percentage (39/50, 78%) of eligible participants enrolled in the study, and we observed a low (1/39, 3%) percentage of loss to follow-up. We had no missing outcome data, and we demonstrated success in our intervention delivery with 100% of SMS text messages being received. Compliance was high, and participants indicated a high degree of satisfaction, motivation, and no difficulties with study instruments, suggesting acceptability of the study. In support of our hypotheses, the intervention program was feasible and acceptable. In this pilot, we further observed a high degree of compliance with the EMI, which showed a promising trend for decreasing sedentary time and a trend for increasing physical activity in the EMI group, compared to the physical activity guidelines group. Although underpowered, we observed moderate effect sizes indicating the plausibility of EMI decreasing sedentary time and replacing it with physical activity. This is in contrast with the study by Hartman et al [11] which reported that a physical activity intervention designed to increase MVPA also significantly increased sedentary time compared to control in Latina women (mean age 39.2, SD 10.33 years) over 6 and 12 months. These authors postulated that the increase in sedentary time was due to compensatory resting after exercise per the “activitystat” hypothesis [12,13]. Balbim et al [44] also reported a decrease in sedentary time in a health education group compared to a dance intervention group, postulated to be due to compensatory resting behavior in the dance group. Other research has reported that physical activity and exercise interventions have little or no impact on sedentary time with 1 meta-analysis by Martin et al [14] showing a sex-specific reduction in sedentary time in males only. In addition, most previous research on sedentary time and physical activity has been conducted in non-Hispanic White populations and has not been designed to replace sedentary time with physical activity during activities of daily living. Our pilot findings may differ due to a shorter intervention period compared to that of Hartman et al [11]. We also included both male and female participants. However, sex-specific analyses did not indicate any differences in outcomes. Our sample was similar in mean age to that of Balbim et al [44], suggesting that age may not have been the contributing factor. It is possible that by focusing on replacing sedentary time with individual physical activity options in real time, we can avoid participants feeling tired from scheduled exercise, and therefore, they are resting less and spending less time being sedentary. This needs to be confirmed in an efficacy study. In addition, by using the principles of the SEM, it is possible that we influenced behavior change by using the interaction between the individual and their living environment. We also included parts of the transtheoretical model by adjusting the EMI based on the stage of behavior change and providing motivational feedback and encouragement via individualized SMS text messages. By providing motivational feedback and outcome successes, we likely improved self-efficacy beliefs as explained by the social cognitive theory; this in turn empowered participants to achieve behavior change. Unfortunately, we did not assess self-efficacy in this study, but it is likely that self-efficacy is a mediating factor, and future studies should investigate this.

Importantly, by replacing sedentary time with a mixture of low-intensity physical activity (+27.6 min), and MVPA (+51.6 min), it is possible that exercise-induced cardiometabolic risk factors decreased [45], albeit due to pandemic restrictions, we were not able to assess these. The physical activity guidelines group decreased MVPA slightly while sedentary time did not change, suggesting that activity guidelines alone will not affect behavior change. In addition, the step count increased by 5542 steps in the intervention group and decreased by 3748 steps in the physical activity guidelines group with a moderate effect size. Our findings of an increase in step count are congruent with the findings reported at 4 weeks and 12 months by Piedra et al [46] who reported an increase in 7-day pedometer step count in a supervised weekly exercise class compared to health education in older Latino adults, albeit we observed a slightly larger increase in daily step count. Of note, our findings conflict with those reported by Menkin et al [47] who found improvement in step count following an 8-session intervention to increase walking in older underrepresented adults at month 1 but not at month 2. It is likely that an 8-session intervention is not intensive enough to instill behavior change and that the use of an EMI program during routine activities of daily living may provide for a more intensive intervention to induce behavior change. Our promising findings of increased MVPA were also congruent with finding by Marquez et al [48] who reported an increase of approximately 156 minutes per week of MVPA (using self-reported physical activity; Community Healthy Activities Model Program for Seniors questionnaire) in older Latino adults randomized to an in-person dance intervention. We observed a slightly smaller increase (51.56 min), likely due to the intervention placing emphasis on replacing sedentary time with physical activity and not exercise per se and delivering the intervention during the course of participant’s daily life. We also used actigraphy measures, compared to self-reported physical activity, which may explain why we observed a slightly larger total activity count using 7-day actigraphy compared to the study by Marquez et al [48]. Importantly, our compliance rate with the intervention was high (79%), suggesting that participants are open to participating in an EMI program.

Although the study was underpowered, we observed a significant increase in executive function as measured by TMT-B time to completion and a reduction in errors when completing the TMT-B in the intervention group compared to the control group. In addition, there were trends for nonsignificant improvements in oral reading recognition and crystallized cognition scores from baseline in the intervention group with modest effect sizes similar to other exercise studies in primarily White populations [43]. These findings are congruent with those of Piedra et al [49] who found improvements in the Mini-Mental State Examination at 1- and 2-year follow-up in older Latino adults randomized to receive a 4-week exercise and attribution retraining program with re-enforcement compared to health education. However, our results differ from those of Aguiñaga et al [50] who did not find an improvement in executive function using the TMT-B in older Latino adults following a 4-month dance intervention, but rather reported an improvement in working memory assessed by the digit span test. We did not observe an improvement in working memory in this study. It is possible that a 6-week intervention is not sufficient for improvement in working memory or global cognition.

These results indicate the plausibility of an individualized EMI program designed for midlife and older Latino adults to be successful in replacing sitting time with light activity and MVPA and improving executive function. These findings need to be confirmed in a larger and fully powered efficacy trial. Exploratory analyses suggest that the observed improvement in executive function could potentially be mediated via a combination of reduced sedentary time and increased MVPA. These findings are important since older Latino adults in the United States will comprise a significant proportion of the overall population in the coming decades. This, in addition to the disproportionate risk for ADRD among this group, presents the need for individually and culturally tailored interventions to promote health and reduce the risk of cognitive decline. These findings need to be assessed in a larger sample designed to test efficacy. Importantly, any physical activity intervention needs to be sustainable in the long term and in the community. Although we did not assess sustainability in this study, it is plausible that an EMI program has the potential to translate into long-term behavior change using the principles of SEM. The results associated with our outcome measures, in conjunction with findings from the participant satisfaction survey, indicate the feasibility, acceptability, and preliminary plausibility of an individualized culturally tailored EMI for improving sedentary behaviors within the Latino population.

### Strengths

The strengths of this study included the randomized controlled design and a high rate of compliance associated with the intervention. This allowed for optimal delivery of motivational messages as well as reliable and consistent data collection via the iCardia platform. This was an entirely virtual intervention suggesting that we can avoid the requirement of in-person attendance, and therefore, increase our ability to reach working adults and adults that may not have the means to participate in-person. The use of the Fitbit activity tracker that is widely available means that the intervention is easily scalable. Our sample is highly representative of the Chicago Latino population, and the results are generalizable [16]. The intervention was culturally and individually tailored and delivered in real time to participants. Developing interventions that have the potential to create a sustainable behavior change in physical activity is important to gain the benefits of moving more and sitting less. Our intervention is dynamic and allows for individual and community factors to achieve sustainable behavior change.

### Limitations

Though several promising results have been produced, this study is not without its limitations. The small sample size and ratio of male-to-female participants reduced the generalizability of our findings. The sample consisted of only midlife and older Latino adults from Chicago. Due to the heterogeneity and intricacies of Latino individuals in the United States, it is possible that Latino individuals in other parts of the United States differ from Latino individuals in Chicago. A multisite comparison effectiveness trial should be conducted. It is time consuming to interview individuals to design an individualized program that may hinder scalability. One plausible direction is to use machine learning to automate ways of personalization. The intervention period was short due to the proof-of-concept nature of the study, but we used alternative forms of cognitive function assessment when available to control possible learning effects. A larger and longer duration study is needed, including a sustainability period. Due to the nature of the pilot study, we were unable to provide Fitbits to the control group, which may have confounded the outcomes due to motivation. In addition, restrictions in place due to the COVID-19 pandemic prevented us from performing additional procedures that could have further validated our findings. For example, we were limited to performing cognitive testing from the NIH Toolbox that was approved for virtual administration only. However, this study would have benefitted from administering motor function testing and magnetic resonance imaging of the brain at baseline and follow-up to fully evaluate changes to functional connectivity networks. In the future, our team plans to improve the representation of the study sample by expanding to include more males. We will also seek to conduct more detailed connectivity analyses, examine the duration of treatment effects over time, and examine mediators and moderators of mechanisms of action.

In sum, this study fills a critical gap in the literature by addressing the need to determine aspects of interventional programs that show promise to fit the needs of the Latino older adult community. The EMI program was feasible and acceptable. This was accomplished through the use of an EMI designed to deliver content in real time, and in the real-world environment. The design and methods of the intervention enabled real-time delivery of behavior options and real-time feedback on the effectiveness of behavior choices. This allowed us to create an individual and culturally tailored intervention that was based on preferences of individuals within their community networks. The results presented in this paper can be used to support the design of future physical activity interventions targeted to forestall or prevent cognitive decline in this population and inform improvements in the design and implementation of meaningful interventions that are culturally appropriate and relevant to the needs of the aging Latino population in the United States. It is especially important to develop physical activity interventions that not only increase physical activity but also reduce sedentary time. These interventions need to be sustainable and targeted to disenfranchised and marginalized populations who may not speak or prefer English and who have disadvantaged socioeconomic status. The EMI program shows promise in meeting this critical need.

### Conclusions

An EMI program designed to replace sedentary time with physical activity is feasible and acceptable. The effect sizes suggest plausibility that a 6-week EMI program can replace sitting time with physical activity and improve executive functioning in older Latino adults. It is plausible that the improvement in executive function is related to a combination of reduced sedentary time and increased MVPA. The clinical implication of our work remains to be further explored.

## Appendix

Multimedia Appendix 1 CONSORT-eHEALTH checklist (V 1.6.1).

## Abbreviations

ADRD: Alzheimer disease and related dementias
CONSORT: Consolidated Standards of Reporting Trials
EMA: ecological momentary assessment
EMI: ecological momentary intervention
MVPA: moderate to vigorous physical activity
NIH: National Institutes of Health
REDCap: Research Electronic Data Capture
SEM: socioecological model
TMT-A: trail making test part A
TMT-B: trail making test part B
