# Supplementary material for: Ecological Momentary Intervention to Replace Sedentary Time With Physical Activity to Improve Executive Function in Midlife and Older Latino Adults: Pilot Randomized Controlled Trial
Source: J Med Internet Res. 2024 Sep 5;26:e55079. doi: 10.2196/55079 (PMC11413544; doi:10.2196/55079)
Supplement: Multimedia Appendix 1 [file jmir_v26i1e55079_app1.pdf]

CONSORT-EHEALTH (V 1.6.1) - Submission/Publication Form

The CONSORT-EHEALTH checklist is intended for authors of randomized trials evaluating web-based and Internet-based applications/interventions, including mobile interventions, electronic games (incl multiplayer games), social media, certain telehealth applications, and other interactive and/or networked electronic applications. Some of the items (e.g. all subitems under item 5 - description of the intervention) may also be applicable for other study designs.

The goal of the CONSORT EHEALTH checklist and guideline is to be
a) a guide for reporting for authors of RCTs,
b) to form a basis for appraisal of an ehealth trial (in terms of validity)

CONSORT-EHEALTH items/subitems are MANDATORY reporting items for studies published in the Journal of Medical Internet Research and other journals / scientific societies endorsing the checklist.

Items numbered 1., 2., 3., 4a., 4b etc are original CONSORT or CONSORT-NPT (non-pharmacologic treatment) items.
Items with Roman numerals (i., ii, iii, iv etc.) are CONSORT-EHEALTH extensions/clarifications.

As the CONSORT-EHEALTH checklist is still considered in a formative stage, we would ask that you also RATE ON A SCALE OF 1-5 how important/useful you feel each item is FOR THE PURPOSE OF THE CHECKLIST and reporting guideline (optional).

Mandatory reporting items are marked with a red *.
In the textboxes, either copy & paste the relevant sections from your manuscript into this form - please include any quotes from your manuscript in QUOTATION MARKS,
or answer directly by providing additional information not in the manuscript, or elaborating on why the item was not relevant for this study.

YOUR ANSWERS WILL BE PUBLISHED AS A SUPPLEMENTARY FILE TO YOUR PUBLICATION IN JMIR AND ARE CONSIDERED PART OF YOUR PUBLICATION (IF ACCEPTED).
Please fill in these questions diligently. Information will not be copyedited, so please use proper spelling and grammar, use correct capitalization, and avoid abbreviations.

DO NOT FORGET TO SAVE AS PDF _AND_ CLICK THE SUBMIT BUTTON SO YOUR ANSWERS ARE IN OUR DATABASE !!!

Citation Suggestion (if you append the pdf as Appendix we suggest to cite this paper in the caption):
Eysenbach G, CONSORT-EHEALTH Group

CONSORT-EHEALTH: Improving and Standardizing Evaluation Reports of Web-based and Mobile Health Interventions
J Med Internet Res 2011;13(4):e126
URL: http://www.jmir.org/2011/4/e126/
doi: 10.2196/jmir.1923
PMID: 22209829

Sign in to Google to save your progress. Learn more

* Indicates required question

Your name *

First Last

Ulf Bronas

Primary Affiliation (short), City, Country *

University of Toronto, Toronto, Canada

Columbia University, New York, USA

Your e-mail address *

abc@gmail.com

ub2154@cumc.columbia.edu

Title of your manuscript *

Provide the (draft) title of your manuscript.

Ecological Momentary Intervention to Replace Sedentary Time with Physical Activity to Improve Executive Function in Mid-life and Older Latino Adults: A randomized controlled trial

Name of your App/Software/Intervention *

If there is a short and a long/alternate name, write the short name first and add the long name in brackets.

Replacing Sedentary Time with Physical Activi

Evaluated Version (if any)

e.g. "V1", "Release 2017-03-01", "Version 2.0.27913"

Your answer

Language(s) *

What language is the intervention/app in? If multiple languages are available, separate by comma (e.g. "English, French")

English

URL of your Intervention Website or App

e.g. a direct link to the mobile app on app in appstore (itunes, Google Play), or URL of the website. If the intervention is a DVD or hardware, you can also link to an Amazon page.

Your answer

URL of an image/screenshot (optional)

Your answer

Accessibility *

Can an enduser access the intervention presently?

- ◯ access is free and open
- ◯ access only for special usergroups, not open
- ◯ access is open to everyone, but requires payment/subscription/in-app purchases
- ◯ app/intervention no longer accessible
- ◉ Other: The intervention is available upon publication of the manuscript as it i

Primary Medical Indication/Disease/Condition *

e.g. "Stress", "Diabetes", or define the target group in brackets after the condition, e.g. "Autism (Parents of children with)", "Alzheimers (Informal Caregivers of)"

Replacing sedentary time with physical activity

Primary Outcomes measured in trial *

comma-separated list of primary outcomes reported in the trial

Sedentary time

Secondary/other outcomes

Are there any other outcomes the intervention is expected to affect?

Physical activity, cognition

Recommended "Dose" *

What do the instructions for users say on how often the app should be used?

- (x) Approximately Daily
- ( ) Approximately Weekly
- ( ) Approximately Monthly
- ( ) Approximately Yearly
- ( ) "as needed"
- ( ) Other:

Approx. Percentage of Users (starters) still using the app as recommended after 3 months *

- (x) unknown / not evaluated
- ( ) 0-10%
- ( ) 11-20%
- ( ) 21-30%
- ( ) 31-40%
- ( ) 41-50%
- ( ) 51-60%
- ( ) 61-70%
- ( ) 71%-80%
- ( ) 81-90%
- ( ) 91-100%
- ( ) Other:

Overall, was the app/intervention effective? *

- (x) yes: all primary outcomes were significantly better in intervention group vs control
- ( ) partly: SOME primary outcomes were significantly better in intervention group vs control
- ( ) no statistically significant difference between control and intervention
- ( ) potentially harmful: control was significantly better than intervention in one or more outcomes
- ( ) inconclusive: more research is needed
- ( ) Other:

Article Preparation Status/Stage *

At which stage in your article preparation are you currently (at the time you fill in this form)

- ( ) not submitted yet - in early draft status
- ( ) not submitted yet - in late draft status, just before submission
- ( ) submitted to a journal but not reviewed yet
- ( ) submitted to a journal and after receiving initial reviewer comments
- (x) submitted to a journal and accepted, but not published yet
- ( ) published
- ( ) Other:

Journal *

If you already know where you will submit this paper (or if it is already submitted), please provide the journal name (if it is not JMIR, provide the journal name under "other")

- ○ not submitted yet / unclear where I will submit this
- ◉ Journal of Medical Internet Research (JMIR)
- ○ JMIR mHealth and UHealth
- ○ JMIR Serious Games
- ○ JMIR Mental Health
- ○ JMIR Public Health
- ○ JMIR Formative Research
- ○ Other JMIR sister journal
- ○ Other:

Is this a full powered effectiveness trial or a pilot/feasibility trial? *

- ◉ Pilot/feasibility
- ○ Fully powered

Manuscript tracking number *

If this is a JMIR submission, please provide the manuscript tracking number under "other" (The ms tracking number can be found in the submission acknowledgement email, or when you login as author in JMIR. If the paper is already published in JMIR, then the ms tracking number is the four-digit number at the end of the DOI, to be found at the bottom of each published article in JMIR)

- ○ no ms number (yet) / not (yet) submitted to / published in JMIR
- ◉ Other: 55079

TITLE AND ABSTRACT

1a) TITLE: Identification as a randomized trial in the title

1a) Does your paper address CONSORT item 1a? *

I.e does the title contain the phrase "Randomized Controlled Trial"? (if not, explain the reason under "other")

- (selected) yes
- ( ) Other:

1a-i) Identify the mode of delivery in the title

Identify the mode of delivery. Preferably use “web-based” and/or “mobile” and/or “electronic game” in the title. Avoid ambiguous terms like “online”, “virtual”, “interactive”. Use “Internet-based” only if Intervention includes non-web-based Internet components (e.g. email), use “computer-based” or “electronic” only if offline products are used. Use “virtual” only in the context of “virtual reality” (3-D worlds). Use “online” only in the context of “online support groups”. Complement or substitute product names with broader terms for the class of products (such as “mobile” or “smart phone” instead of “iphone”), especially if the application runs on different platforms.

	1	2	3	4	5	
subitem not at all important	( )	(selected)	( )	( )	( )	essential

Clear selection

Does your paper address subitem 1a-i? *

Copy and paste relevant sections from manuscript title (include quotes in quotation marks "like this" to indicate direct quotes from your manuscript), or elaborate on this item by providing additional information not in the ms, or briefly explain why the item is not applicable/relevant for your study

Addressed as Ecological Momentary Intervention. The title would be much too long otherwise.

1a-ii) Non-web-based components or important co-interventions in title

Mention non-web-based components or important co-interventions in title, if any (e.g., “with telephone support”).

	1	2	3	4	5	
subitem not at all important	○	●	○	○	○	essential

Clear selection

Does your paper address subitem 1a-ii?

Copy and paste relevant sections from manuscript title (include quotes in quotation marks "like this" to indicate direct quotes from your manuscript), or elaborate on this item by providing additional information not in the ms, or briefly explain why the item is not applicable/relevant for your study

No. The title would be much too long.

1a-iii) Primary condition or target group in the title

Mention primary condition or target group in the title, if any (e.g., “for children with Type I Diabetes”) Example: A Web-based and Mobile Intervention with Telephone Support for Children with Type I Diabetes: Randomized Controlled Trial

	1	2	3	4	5	
subitem not at all important	○	○	○	○	◉	essential

Clear selection

Does your paper address subitem 1a-iii? *

Copy and paste relevant sections from manuscript title (include quotes in quotation marks "like this" to indicate direct quotes from your manuscript), or elaborate on this item by providing additional information not in the ms, or briefly explain why the item is not applicable/relevant for your study

Target sample is essential and addressed in the title. Mid-life and older Latino Adults

1b) ABSTRACT: Structured summary of trial design, methods, results, and conclusions

NPT extension: Description of experimental treatment, comparator, care providers, centers, and blinding status.

1b-i) Key features/functionalities/components of the intervention and comparator in the METHODS section of the ABSTRACT

Mention key features/functionalities/components of the intervention and comparator in the abstract. If possible, also mention theories and principles used for designing the site. Keep in mind the needs of systematic reviewers and indexers by including important synonyms. (Note: Only report in the abstract what the main paper is reporting. If this information is missing from the main body of text, consider adding it)

	1	2	3	4	5	
subitem not at all important	○	○	◉	○	○	essential

Clear selection

Does your paper address subitem 1b-i? *

Copy and paste relevant sections from the manuscript abstract (include quotes in quotation marks "like this" to indicate direct quotes from your manuscript), or elaborate on this item by providing additional information not in the ms, or briefly explain why the item is not applicable/relevant for your study

Objective: To develop and test an Ecological Momentary Intervention (EMI) that is culturally and individually designed to replace sedentary time with physical activity in mid-life and older Latinos.
Methods:
This study randomized mid-life and older Spanish-speaking Latinos to either a 6-week EMI program designed to replace sitting time with physical activity, or to a group that received education on physical activity guidelines. The program was conducted virtually and in Spanish. The intervention was individualized based on individual interview responses to ensure that it was culturally appropriate. The intervention included use of a Fitbit activity monitor and mobile app, weekly didactic phone meetings, interactive tools (e.g., text messages/app reminders), and coach-delivered feedback. Sedentary time and physical activity were assessed via 7-day actigraphy worn on the hip. Cognitive performance was assessed via the trail making test part A and B (executive function), and via the NIH Toolbox remote cognitive assessment. Statistical analysis included linear model on change score from baseline, adjusting for age, sex, and education.

1b-ii) Level of human involvement in the METHODS section of the ABSTRACT

Clarify the level of human involvement in the abstract, e.g., use phrases like “fully automated” vs. “therapist/nurse/care provider/physician-assisted” (mention number and expertise of providers involved, if any). (Note: Only report in the abstract what the main paper is reporting. If this information is missing from the main body of text, consider adding it)

	1	2	3	4	5	
subitem not at all important	○	○	◉	○	○	essential

Clear selection

Does your paper address subitem 1b-ii?

Copy and paste relevant sections from the manuscript abstract (include quotes in quotation marks "like this" to indicate direct quotes from your manuscript), or elaborate on this item by providing additional information not in the ms, or briefly explain why the item is not applicable/relevant for your study

The intervention included use of a Fitbit activity monitor and mobile app, weekly didactic phone meetings, interactive tools (e.g., text messages/app reminders), and coach-delivered feedback.

1b-iii) Open vs. closed, web-based (self-assessment) vs. face-to-face assessments in the METHODS section of the ABSTRACT

Mention how participants were recruited (online vs. offline), e.g., from an open access website or from a clinic or a closed online user group (closed usergroup trial), and clarify if this was a purely web-based trial, or there were face-to-face components (as part of the intervention or for assessment). Clearly say if outcomes were self-assessed through questionnaires (as common in web-based trials). Note: In traditional offline trials, an open trial (open-label trial) is a type of clinical trial in which both the researchers and participants know which treatment is being administered. To avoid confusion, use "blinded" or "unblinded" to indicated the level of blinding instead of "open", as "open" in web-based trials usually refers to "open access" (i.e. participants can self-enrol). (Note: Only report in the abstract what the main paper is reporting. If this information is missing from the main body of text, consider adding it)

	1	2	3	4	5	
subitem not at all important	○	○	●	○	○	essential

Clear selection

Does your paper address subitem 1b-iii?

Copy and paste relevant sections from the manuscript abstract (include quotes in quotation marks "like this" to indicate direct quotes from your manuscript), or elaborate on this item by providing additional information not in the ms, or briefly explain why the item is not applicable/relevant for your study

community-dwelling recruited in the community. Physical activity intervention. PI blinded to group assignment.

1b-iv) RESULTS section in abstract must contain use data

Report number of participants enrolled/assessed in each group, the use/uptake of the intervention (e.g., attrition/adherence metrics, use over time, number of logins etc.), in addition to primary/secondary outcomes. (Note: Only report in the abstract what the main paper is reporting. If this information is missing from the main body of text, consider adding it)

	1	2	3	4	5	
subitem not at all important	○	○	◉	○	○	essential

Clear selection

Does your paper address subitem 1b-iv?

Copy and paste relevant sections from the manuscript abstract (include quotes in quotation marks "like this" to indicate direct quotes from your manuscript), or elaborate on this item by providing additional information not in the ms, or briefly explain why the item is not applicable/relevant for your study

A total of 39 mid-life and older Spanish-speaking Latinos were randomized (26 female, mean age 61.0 (5.8)). The intervention group had a 79% compliance rate to the intervention. Trail making test part B time to completion and number of errors improved over time in the intervention group (-35.26 + 60.35 seconds and -1.98 + 2.19 respectively), compared to the control group (7.19 + 46 seconds and 0.37 +2.24 respectively, d=.74, p=.006). Weekly step count increased in the intervention group by 5,543 steps (d=.54, p=.052) and sedentary time decreased by 348 +485 minutes (d=.47, p=.24), compared to control. Percent of time spent performing moderately-high intensity activity increased in the intervention group, compared to the control group (d=.67, p=.015). Participant satisfaction was high and a high degree of motivation to replace sitting time with physical activity was reported.

1b-v) CONCLUSIONS/DISCUSSION in abstract for negative trials

Conclusions/Discussions in abstract for negative trials: Discuss the primary outcome - if the trial is negative (primary outcome not changed), and the intervention was not used, discuss whether negative results are attributable to lack of uptake and discuss reasons. (Note: Only report in the abstract what the main paper is reporting. If this information is missing from the main body of text, consider adding it)

	1	2	3	4	5	
subitem not at all important	○	○	●	○	○	essential

Clear selection

Does your paper address subitem 1b-v?

Copy and paste relevant sections from the manuscript abstract (include quotes in quotation marks "like this" to indicate direct quotes from your manuscript), or elaborate on this item by providing additional information not in the ms, or briefly explain why the item is not applicable/relevant for your study

An individualized ecological momentary intervention designed for mid-life and older Latinos was successful in replacing sitting time with physical activity and improved executive functioning. The intervention was feasible and well received with a high degree of satisfaction.

INTRODUCTION

2a) In INTRODUCTION: Scientific background and explanation of rationale

2a-i) Problem and the type of system/solution

Describe the problem and the type of system/solution that is object of the study: intended as stand-alone intervention vs. incorporated in broader health care program? Intended for a particular patient population? Goals of the intervention, e.g., being more cost-effective to other interventions, replace or complement other solutions? (Note: Details about the intervention are provided in “Methods” under 5)

	1	2	3	4	5	
subitem not at all important	○	○	◉	○	○	essential

Clear selection

Does your paper address subitem 2a-i? *

Copy and paste relevant sections from the manuscript (include quotes in quotation marks "like this" to indicate direct quotes from your manuscript), or elaborate on this item by providing additional information not in the ms, or briefly explain why the item is not applicable/relevant for your study

The current view of the field is rooted in a Eurocentric view, which assumes that interventions that work in one community will work the same in a different community. This assumption is likely incorrect and represents a significant gap in our ability to develop a successful intervention at the multiple levels that are required across the socio-ecological model (SEM) to be successful23,24. Thus, developing and testing the effect of a culturally appropriate and community based ecological momentary intervention (EMI, delivered in real-time during participants everyday lives) on replacing sedentary time with physical activity, and cognitive performance is warranted. Additionally, including participants in that latter part of mid-life is tremendously important since this represents a window of opportunity for behavior change to allow for healthy aging. Due to the heterogeneity and intricacies of mid-life and older Latinos in the Chicago area and to ensure cultural validity of the EMI, we need to understand factors that facilitate initiation of and adherence to behavioral change at the individual level using the social cognitive theory. 25-27.
Therefore, the present study aimed to determine the feasibility, acceptability, and effect of a 6-week ecological momentary intervention program on sedentary time, daily physical activity, and cognitive performance in mid-life and older Latinos, delivered in the home-setting over 6 weeks, compared to participants randomized to receive physical activity guidelines in Spanish. We hypothesized that the EMI program would result in (a) reduced sedentary time (sitting, primary outcome), (b) increased device assessed daily physical activity (secondary outcome), and (c) improved cognitive performance (executive functioning and cognitive flexibility, secondary outcome), compared to a group randomized to receive education on physical activity guidelines.

2a-ii) Scientific background, rationale: What is known about the (type of) system

Scientific background, rationale: What is known about the (type of) system that is the object of the study (be sure to discuss the use of similar systems for other conditions/diagnoses, if appropiate), motivation for the study, i.e. what are the reasons for and what is the context for this specific study, from which stakeholder viewpoint is the study performed, potential impact of findings [2]. Briefly justify the choice of the comparator.

	1	2	3	4	5	
subitem not at all important	○	○	◉	○	○	essential

Clear selection

Does your paper address subitem 2a-ii? *

Copy and paste relevant sections from the manuscript (include quotes in quotation marks "like this" to indicate direct quotes from your manuscript), or elaborate on this item by providing additional information not in the ms, or briefly explain why the item is not applicable/relevant for your study

The United States population of adults aged 65 and older is projected to increase from 58 million in 2021 to approximately 88 million by the year 20501. Concurrently, it is estimated that 13.8 million Americans will be diagnosed with Alzheimer's Disease or Related Dementias (ADRD) by that same year2. This is a significant increase from the approximately 5 million diagnosed with ADRD in 20143. The Latino/a/x/e (hereafter referred to as Latino) population within this age cohort will comprise 18% of the total U.S. population by 20504. The growth projected within this subgroup has significant implications for the burden of ADRD, as it has been well established that rates of ADRD are disproportionately higher among older Latino adults compared to other ethnicities of the same age1,4-6. Currently, over 12% of older Latinos have been diagnosed with ADRD6. Even more striking, the number of Latinos with ADRD is expected to increase 7-fold over the next three decades, representing an epidemic increase of ADRD3. The high incidence of ADRD in conjunction with physical function limitations place older Latinos at a high risk for loss of independence and significant caregiver burden.
Additionally, older Latinos have a higher prevalence of risk factors for cognitive decline and the rate of increase in risk factors is greatest among Latinos.7,8 Latinos further engage in less leisure time physical activity and spend more time sedentary, compared to non-Latino White participants, ranging from 46.8-74.2% Vs. 34.3-58.2%.9,10 Thus, increasing physical activity is of tremendous significance to this population. Of note, physical activity programs that have successfully increased daily physical activity in older Latinos often report an increase in sedentary time.11-15 Sedentary time, defined as not raising energy expenditure above 1.5 METs and includes lying down, sitting, watching television, and other screen-based entertainment activities, is a known risk factor for reduced cognitive function, especially executive function, in older Latinos16-19. Thus, studies are clearly needed to investigate the benefits of breaking up and replacing sedentary time with physical activity (PA) on cognition and brain connectivity in older Latinos20-22. However, to develop and deliver a successful intervention we first need to understand how to best conduct a lifestyle intervention to replace sitting time with physical activity in the Latino community.
The current view of the field is rooted in a Eurocentric view, which assumes that interventions that work in one community will work the same in a different community. This assumption is likely incorrect and represents a significant gap in our ability to develop a successful intervention at the multiple levels that are required across the socio-ecological model (SEM) to be successful23,24. Thus, developing and testing the effect of a culturally appropriate and community based ecological momentary intervention (EMI, delivered in real-time during participants everyday lives) on replacing sedentary time with physical activity, and cognitive performance is warranted. Additionally, including participants in that latter part of mid-life is tremendously important since this represents a window of opportunity for behavior change to allow for healthy aging. Due to the heterogeneity and intricacies of mid-life and older Latinos in the Chicago area and to ensure cultural validity of the EMI, we need to understand factors that facilitate initiation of and adherence to behavioral change at the individual level using the social cognitive theory. 25-27.
Therefore, the present study aimed to determine the feasibility, acceptability, and effect of a 6-week ecological momentary intervention program on sedentary time, daily physical activity, and cognitive performance in mid-life and older Latinos, delivered in the home-setting over 6 weeks, compared to participants randomized to receive physical activity guidelines in Spanish. We hypothesized that the EMI program would result in (a) reduced sedentary time (sitting, primary outcome), (b) increased device assessed daily physical activity (secondary outcome), and (c) improved cognitive performance (executive functioning and cognitive flexibility, secondary outcome), compared to a group randomized [illegible]

functioning and cognitive flexibility, secondary outcome), compared to a group randomized to receive education on physical activity guidelines.

2b) In INTRODUCTION: Specific objectives or hypotheses

Does your paper address CONSORT subitem 2b? *

Copy and paste relevant sections from the manuscript (include quotes in quotation marks "like this" to indicate direct quotes from your manuscript), or elaborate on this item by providing additional information not in the ms, or briefly explain why the item is not applicable/relevant for your study

Therefore, the present study aimed to determine the feasibility, acceptability, and effect of a 6-week ecological momentary intervention program on sedentary time, daily physical activity, and cognitive performance in mid-life and older Latinos, delivered in the home-setting over 6 weeks, compared to participants randomized to receive physical activity guidelines in Spanish. We hypothesized that the EMI program would result in (a) reduced sedentary time (sitting, primary outcome), (b) increased device assessed daily physical activity (secondary outcome), and (c) improved cognitive performance (executive functioning and cognitive flexibility, secondary outcome), compared to a group randomized to receive education on physical activity guidelines.

METHODS

3a) Description of trial design (such as parallel, factorial) including allocation ratio

Does your paper address CONSORT subitem 3a? *

Copy and paste relevant sections from the manuscript (include quotes in quotation marks "like this" to indicate direct quotes from your manuscript), or elaborate on this item by providing additional information not in the ms, or briefly explain why the item is not applicable/relevant for your study

This study was a pilot of a two-armed, randomized controlled trial.

3b) Important changes to methods after trial commencement (such as eligibility criteria), with reasons

Does your paper address CONSORT subitem 3b? *

Copy and paste relevant sections from the manuscript (include quotes in quotation marks "like this" to indicate direct quotes from your manuscript), or elaborate on this item by providing additional information not in the ms, or briefly explain why the item is not applicable/relevant for your study

No changes were made to the trial design or conduct after initiation of study

3b-i) Bug fixes, Downtimes, Content Changes

Bug fixes, Downtimes, Content Changes: ehealth systems are often dynamic systems. A description of changes to methods therefore also includes important changes made on the intervention or comparator during the trial (e.g., major bug fixes or changes in the functionality or content) (5-iii) and other “unexpected events” that may have influenced study design such as staff changes, system failures/downtimes, etc. [2].

	1	2	3	4	5	
subitem not at all important	○	○	●	○	○	essential

Clear selection

Does your paper address subitem 3b-i?

Copy and paste relevant sections from the manuscript (include quotes in quotation marks "like this" to indicate direct quotes from your manuscript), or elaborate on this item by providing additional information not in the ms, or briefly explain why the item is not applicable/relevant for your study

No changes were made and no bugs were discovered in the study.

4a) Eligibility criteria for participants

Does your paper address CONSORT subitem 4a? *

Copy and paste relevant sections from the manuscript (include quotes in quotation marks "like this" to indicate direct quotes from your manuscript), or elaborate on this item by providing additional information not in the ms, or briefly explain why the item is not applicable/relevant for your study

Ultimately, 69 participants agreed to be assessed for eligibility. Inclusion criteria were (a) 55-89 years of age, (b) no major head trauma, or (c) ownership of a working smartphone with internet/messaging plan. Exclusion Criteria: Participating in a supervised exercise program, wheelchair dependent, diagnosed dementia, medications to improve cognition or mood; contraindications to PA per ACSM guidelines.28

4a-i) Computer / Internet literacy

Computer / Internet literacy is often an implicit “de facto” eligibility criterion - this should be explicitly clarified.

	1	2	3	4	5	
subitem not at all important	○	○	●	○	○	essential

Clear selection

Does your paper address subitem 4a-i?

Copy and paste relevant sections from the manuscript (include quotes in quotation marks "like this" to indicate direct quotes from your manuscript), or elaborate on this item by providing additional information not in the ms, or briefly explain why the item is not applicable/relevant for your study

Owner of smartphone was inclusion crieteria

4a-ii) Open vs. closed, web-based vs. face-to-face assessments:

Open vs. closed, web-based vs. face-to-face assessments: Mention how participants were recruited (online vs. offline), e.g., from an open access website or from a clinic, and clarify if this was a purely web-based trial, or there were face-to-face components (as part of the intervention or for assessment), i.e., to what degree got the study team to know the participant. In online-only trials, clarify if participants were quasi-anonymous and whether having multiple identities was possible or whether technical or logistical measures (e.g., cookies, email confirmation, phone calls) were used to detect/prevent these.

	1	2	3	4	5	
subitem not at all important	○	○	◉	○	○	essential

Clear selection

Does your paper address subitem 4a-ii? *

Copy and paste relevant sections from the manuscript (include quotes in quotation marks "like this" to indicate direct quotes from your manuscript), or elaborate on this item by providing additional information not in the ms, or briefly explain why the item is not applicable/relevant for your study

We recruited participants through flyers posted at local Latino based organizations and on websites, as well as using word of mouth, and through media advertisements. Investigators also gave presentations at several primarily Spanish-speaking churches to recruit additional participants in the Chicago area

4a-iii) Information giving during recruitment

Information given during recruitment. Specify how participants were briefed for recruitment and in the informed consent procedures (e.g., publish the informed consent documentation as appendix, see also item X26), as this information may have an effect on user self-selection, user expectation and may also bias results.

	1	2	3	4	5	
subitem not at all important	(•)	( )	( )	( )	( )	essential

Clear selection

Does your paper address subitem 4a-iii?

Copy and paste relevant sections from the manuscript (include quotes in quotation marks "like this" to indicate direct quotes from your manuscript), or elaborate on this item by providing additional information not in the ms, or briefly explain why the item is not applicable/relevant for your study

Informed consent and study protocol listed in clinical trials. gov

4b) Settings and locations where the data were collected

Does your paper address CONSORT subitem 4b? *

Copy and paste relevant sections from the manuscript (include quotes in quotation marks "like this" to indicate direct quotes from your manuscript), or elaborate on this item by providing additional information not in the ms, or briefly explain why the item is not applicable/relevant for your study

Participants were first screened to determine eligibility via telephone and those that met criteria completed the informed e-consent conducted via Zoom video. Once consent was obtained, participants provided basic demographic information (age, sex, marital status, ethnicity, preferred language, and education) and a full medical history. We then completed the Charlson co-morbidity questionnaire for each participant and conducted the following NIH Toolbox cognitive function measures as approved by the NIH for use via video call:29 Picture vocabulary test, Picture sequence test, List sorting test, Oral reading recognition test, Auditory verbal learning test, and the Trail Making test A and B (TMT-A, and TMT-B)30-32. TMT-B time to completion was considered the measure of executive function.

4b-i) Report if outcomes were (self-)assessed through online questionnaires

Clearly report if outcomes were (self-)assessed through online questionnaires (as common in web-based trials) or otherwise.

	1	2	3	4	5	
subitem not at all important	○	○	●	○	○	essential

Clear selection

Does your paper address subitem 4b-i? *

Copy and paste relevant sections from the manuscript (include quotes in quotation marks "like this" to indicate direct quotes from your manuscript), or elaborate on this item by providing additional information not in the ms, or briefly explain why the item is not applicable/relevant for your study

Participants were first screened to determine eligibility via telephone and those that met criteria completed the informed e-consent conducted via Zoom video. Once consent was obtained, participants provided basic demographic information (age, sex, marital status, ethnicity, preferred language, and education) and a full medical history. We then completed the Charlson co-morbidity questionnaire for each participant and conducted the following NIH Toolbox cognitive function measures as approved by the NIH for use via video call:29 Picture vocabulary test, Picture sequence test, List sorting test, Oral reading recognition test, Auditory verbal learning test, and the Trail Making test A and B (TMT-A, and TMT-B)30-32. TMT-B time to completion was considered the measure of executive function. Following cognitive function testing, we conducted a video interview, which was recorded, transcribed and translated, for the development of a culturally and individually tailored EMI program (see intervention). Once participants completed the interview portion, they were provided with a triaxial accelerometer (ActiGraph GT3X+, Actigraph, Pensacola, Florida), which they were instructed to wear on their right hip/waist for 7 consecutive days to measure sedentary time and daily physical activity. Upon return of the ActiGraph, participants were randomized and notified of their assigned group (EMI or physical activity guideline education). The accelerometer was worn by both groups at baseline and at follow up. Data collectors instructed the participants on the use do the accelerometer and the use of an accelerometer log.

4b-ii) Report how institutional affiliations are displayed

Report how institutional affiliations are displayed to potential participants [on ehealth media], as affiliations with prestigious hospitals or universities may affect volunteer rates, use, and reactions with regards to an intervention.(Not a required item – describe only if this may bias results)

	1	2	3	4	5	
subitem not at all important	○	○	●	○	○	essential

Clear selection

Does your paper address subitem 4b-ii?

Copy and paste relevant sections from the manuscript (include quotes in quotation marks "like this" to indicate direct quotes from your manuscript), or elaborate on this item by providing additional information not in the ms, or briefly explain why the item is not applicable/relevant for your study

Your answer

5) The interventions for each group with sufficient details to allow replication, including how and when they were actually administered

5-i) Mention names, credential, affiliations of the developers, sponsors, and owners

Mention names, credential, affiliations of the developers, sponsors, and owners [6] (if authors/evaluators are owners or developer of the software, this needs to be declared in a “Conflict of interest” section or mentioned elsewhere in the manuscript).

	1	2	3	4	5	
subitem not at all important	○	○	●	○	○	essential

Clear selection

Does your paper address subitem 5-i?

Copy and paste relevant sections from the manuscript (include quotes in quotation marks "like this" to indicate direct quotes from your manuscript), or elaborate on this item by providing additional information not in the ms, or briefly explain why the item is not applicable/relevant for your study

The EMI program was developed based on the principles of the SEM, by understanding and considering participants' cultural and individual interaction with their living environment.37 We further individualized and adjusted the program based on participants stage of behavior change using the transtheoretical model using text messages designed in part with participant input that included health outcomes. The EMI program was also designed to provide motivational feedback of successes and encouragement via individualized text messages to empower participants in their own ability to achieve behavior change and gain positive results as explained by the social cognitive theory (SCT).38 We achieved this by interviewing each participant. Participants told investigators what times during the day they wanted the text message reminders and how the text messages should be phrased and what they preferred to do for physical activity based on their living-environment. They also provided their preference for which health benefit reminders to be sent and they helped design these to match each individual preference. Participants further told investigators what type of feedback they preferred and when they preferred the delivery of text message feedback of successes.
Participants in the EMI group were sent a Fitbit activity monitor and a research assistant helped them to set it up and configure it to receive sedentary behavior notifications in the form of vibrations when participant walked <250 steps per hour during waking hours (selected by the participant). We then scheduled a video call to set up a HIPPA compliant EMA system (Ilumivu mEMA) and to provide education about the Fitbit and the intervention to disrupt sedentary time. Through use of the Fitbit activity tracker, and the Ilumivu mEMA smart phone app, we were able to implement real-time delivery of the behavior options and feedback on the effectiveness of their behavior choices39 Although the intervention was guided by the individual interview findings, in general, participants received suggestions on their smartphone on how to replace sitting time with PA such as standing up 5 times or taking 20 steps, or even performing a short (20 seconds) preferred dance routine. Participants received reminders from their smartphones to enter real-time feedback on activity options selected and how successful they were in adopting the option. These data allowed us to track underlying preferences for behaviors and tailor the program accordingly to each individual.

5-ii) Describe the history/development process

Describe the history/development process of the application and previous formative evaluations (e.g., focus groups, usability testing), as these will have an impact on adoption/use rates and help with interpreting results.

	1	2	3	4	5	
subitem not at all important	○	○	◉	○	○	essential

Clear selection

Does your paper address subitem 5-ii?

Copy and paste relevant sections from the manuscript (include quotes in quotation marks "like this" to indicate direct quotes from your manuscript), or elaborate on this item by providing additional information not in the ms, or briefly explain why the item is not applicable/relevant for your study

We achieved this by interviewing each participant. Participants told investigators what times during the day they wanted the text message reminders and how the text messages should be phrased and what they preferred to do for physical activity based on their living-environment. They also provided their preference for which health benefit reminders to be sent and they helped design these to match each individual preference. Participants further told investigators what type of feedback they preferred and when they preferred the delivery of text message feedback of successes.

5-iii) Revisions and updating

Revisions and updating. Clearly mention the date and/or version number of the application/intervention (and comparator, if applicable) evaluated, or describe whether the intervention underwent major changes during the evaluation process, or whether the development and/or content was “frozen” during the trial. Describe dynamic components such as news feeds or changing content which may have an impact on the replicability of the intervention (for unexpected events see item 3b).

	1	2	3	4	5	
subitem not at all important	(●)	( )	( )	( )	( )	essential

Clear selection

Does your paper address subitem 5-iii?

Copy and paste relevant sections from the manuscript (include quotes in quotation marks "like this" to indicate direct quotes from your manuscript), or elaborate on this item by providing additional information not in the ms, or briefly explain why the item is not applicable/relevant for your study

There were no changes in the intervention for this study.

5-iv) Quality assurance methods

Provide information on quality assurance methods to ensure accuracy and quality of information provided [1], if applicable.

	1	2	3	4	5	
subitem not at all important	( )	( )	(●)	( )	( )	essential

Clear selection

Does your paper address subitem 5-iv?

Copy and paste relevant sections from the manuscript (include quotes in quotation marks "like this" to indicate direct quotes from your manuscript), or elaborate on this item by providing additional information not in the ms, or briefly explain why the item is not applicable/relevant for your study

Participants received a message with success of positive choices for behavioral action. The resulting actions and delivery of the intervention were automatically captured and downloaded in real time using iCardia and the Ilumivu mEMA smart phone app. Compliance to the EMI was considered <70% of time spent sedentary or a mean of > than 150 minutes of moderate physical activity/week as measured by the Fitbit (activity minutes).

5-v) Ensure replicability by publishing the source code, and/or providing screenshots/screen-capture video, and/or providing flowcharts of the algorithms used

Ensure replicability by publishing the source code, and/or providing screenshots/screen-capture video, and/or providing flowcharts of the algorithms used. Replicability (i.e., other researchers should in principle be able to replicate the study) is a hallmark of scientific reporting.

	1	2	3	4	5	
subitem not at all important	○	○	●	○	○	essential

Clear selection

Does your paper address subitem 5-v?

Copy and paste relevant sections from the manuscript (include quotes in quotation marks "like this" to indicate direct quotes from your manuscript), or elaborate on this item by providing additional information not in the ms, or briefly explain why the item is not applicable/relevant for your study

This is not applicable to this study.

5-vi) Digital preservation

Digital preservation: Provide the URL of the application, but as the intervention is likely to change or disappear over the course of the years; also make sure the intervention is archived (Internet Archive, webcitation.org, and/or publishing the source code or screenshots/videos alongside the article). As pages behind login screens cannot be archived, consider creating demo pages which are accessible without login.

	1	2	3	4	5	
subitem not at all important	○	○	◉	○	○	essential

Clear selection

Does your paper address subitem 5-vi?

Copy and paste relevant sections from the manuscript (include quotes in quotation marks "like this" to indicate direct quotes from your manuscript), or elaborate on this item by providing additional information not in the ms, or briefly explain why the item is not applicable/relevant for your study

This is not applicable to this study

5-vii) Access

Access: Describe how participants accessed the application, in what setting/context, if they had to pay (or were paid) or not, whether they had to be a member of specific group. If known, describe how participants obtained “access to the platform and Internet” [1]. To ensure access for editors/reviewers/readers, consider to provide a “backdoor” login account or demo mode for reviewers/readers to explore the application (also important for archiving purposes, see vi).

	1	2	3	4	5	
subitem not at all important	○	○	◉	○	○	essential

Clear selection

Does your paper address subitem 5-vii? *

Copy and paste relevant sections from the manuscript (include quotes in quotation marks "like this" to indicate direct quotes from your manuscript), or elaborate on this item by providing additional information not in the ms, or briefly explain why the item is not applicable/relevant for your study

Participants in the EMI group were sent a Fitbit activity monitor and a research assistant helped them to set it up and configure it to receive sedentary behavior notifications in the form of vibrations when participant walked <250 steps per hour during waking hours (selected by the participant). We then scheduled a video call to set up a HIPPA compliant EMA system (Ilumivu mEMA) and to provide education about the Fitbit and the intervention to disrupt sedentary time. Through use of the Fitbit activity tracker, and the Ilumivu mEMA smart phone app, we were able to implement real-time delivery of the behavior options and feedback on the effectiveness of their behavior choices39 Although the intervention was guided by the individual interview findings, in general, participants received suggestions on their smartphone on how to replace sitting time with PA such as standing up 5 times or taking 20 steps, or even performing a short (20 seconds) preferred dance routine. Participants received reminders from their smartphones to enter real-time feedback on activity options selected and how successful they were in adopting the option. These data allowed us to track underlying preferences for behaviors and tailor the program accordingly to each individual.

5-viii) Mode of delivery, features/functionalities/components of the intervention and comparator, and the theoretical framework

Describe mode of delivery, features/functionalities/components of the intervention and comparator, and the theoretical framework [6] used to design them (instructional strategy [1], behaviour change techniques, persuasive features, etc., see e.g., [7, 8] for terminology). This includes an in-depth description of the content (including where it is coming from and who developed it) [1],” whether [and how] it is tailored to individual circumstances and allows users to track their progress and receive feedback” [6]. This also includes a description of communication delivery channels and – if computer-mediated communication is a component – whether communication was synchronous or asynchronous [6]. It also includes information on presentation strategies [1], including page design principles, average amount of text on pages, presence of hyperlinks to other resources, etc. [1].

	1	2	3	4	5	
subitem not at all important	○	○	●	○	○	essential

Clear selection

Does your paper address subitem 5-viii? *

Copy and paste relevant sections from the manuscript (include quotes in quotation marks "like this" to indicate direct quotes from your manuscript), or elaborate on this item by providing additional information not in the ms, or briefly explain why the item is not applicable/relevant for your study

The EMI program was developed based on the principles of the SEM, by understanding and considering participants' cultural and individual interaction with their living environment.37 We further individualized and adjusted the program based on participants stage of behavior change using the transtheoretical model using text messages designed in part with participant input that included health outcomes. The EMI program was also designed to provide motivational feedback of successes and encouragement via individualized text messages to empower participants in their own ability to achieve behavior change and gain positive results as explained by the social cognitive theory (SCT).38 We achieved this by interviewing each participant. Participants told investigators what times during the day they wanted the text message reminders and how the text messages should be phrased and what they preferred to do for physical activity based on their living-environment. They also provided their preference for which health benefit reminders to be sent and they helped design these to match each individual preference. Participants further told investigators what type of feedback they preferred and when they preferred the delivery of text message feedback of successes.

Participants in the EMI group were sent a Fitbit activity monitor and a research assistant helped them to set it up and configure it to receive sedentary behavior notifications in the form of vibrations when participant walked <250 steps per hour during waking hours (selected by the participant). We then scheduled a video call to set up a HIPPA compliant EMA system (Ilumivu mEMA) and to provide education about the Fitbit and the intervention to disrupt sedentary time. Through use of the Fitbit activity tracker, and the Ilumivu mEMA smart phone app, we were able to implement real-time delivery of the behavior options and feedback on the effectiveness of their behavior choices39 Although the intervention was guided by the individual interview findings, in general, participants received suggestions on their smartphone on how to replace sitting time with PA such as standing up 5 times or taking 20 steps, or even performing a short (20 seconds) preferred dance routine.

Participants received reminders from their smartphones to enter real-time feedback on activity options selected and how successful they were in adopting the option. These data allowed us to track underlying preferences for behaviors and tailor the program accordingly to each individual.

Participants received a message with success of positive choices for behavioral action. The resulting actions and delivery of the intervention were automatically captured and downloaded in real time using iCardia and the Ilumivu mEMA smart phone app. Compliance to the EMI was considered <70% of time spent sedentary or a mean of > than 150 minutes of moderate physical activity/week as measured by the Fitbit (activity minutes). Participants randomized to the control group were sent and received an hour-long video education on general physical activity guidelines (questions were entertained and answered). At the completion of the 6-week intervention period all participants were again sent an ActiGraph to be worn on the waist for 7 days to measure sedentary time and daily physical activity. Once the ActiGraph was returned, a phone interview was scheduled with each participant to understand where they had difficulty and what aspects of the program did or did not work for them. The cognitive function tests administered at baseline were then repeated.

5-ix) Describe use parameters

Describe use parameters (e.g., intended “doses” and optimal timing for use). Clarify what instructions or recommendations were given to the user, e.g., regarding timing, frequency, heaviness of use, if any, or was the intervention used ad libitum.

	1	2	3	4	5	
subitem not at all important	○	○	◉	○	○	essential

Clear selection

Does your paper address subitem 5-ix?

Copy and paste relevant sections from the manuscript (include quotes in quotation marks "like this" to indicate direct quotes from your manuscript), or elaborate on this item by providing additional information not in the ms, or briefly explain why the item is not applicable/relevant for your study

The EMI program was developed based on the principles of the SEM, by understanding and considering participants' cultural and individual interaction with their living environment.37 We further individualized and adjusted the program based on participants stage of behavior change using the transtheoretical model using text messages designed in part with participant input that included health outcomes. The EMI program was also designed to provide motivational feedback of successes and encouragement via individualized text messages to empower participants in their own ability to achieve behavior change and gain positive results as explained by the social cognitive theory (SCT).38 We achieved this by interviewing each participant. Participants told investigators what times during the day they wanted the text message reminders and how the text messages should be phrased and what they preferred to do for physical activity based on their living-environment. They also provided their preference for which health benefit reminders to be sent and they helped design these to match each individual preference. Participants further told investigators what type of feedback they preferred and when they preferred the delivery of text message feedback of successes.
Participants in the EMI group were sent a Fitbit activity monitor and a research assistant helped them to set it up and configure it to receive sedentary behavior notifications in the form of vibrations when participant walked <250 steps per hour during waking hours (selected by the participant). We then scheduled a video call to set up a HIPPA compliant EMA system (Ilumivu mEMA) and to provide education about the Fitbit and the intervention to disrupt sedentary time. Through use of the Fitbit activity tracker, and the Ilumivu mEMA smart phone app, we were able to implement real-time delivery of the behavior options and feedback on the effectiveness of their behavior choices39 Although the intervention was guided by the individual interview findings, in general, participants received suggestions on their smartphone on how to replace sitting time with PA such as standing up 5 times or taking 20 steps, or even performing a short (20 seconds) preferred dance routine. Participants received reminders from their smartphones to enter real-time feedback on activity options selected and how successful they were in adopting the option. These data allowed us to track underlying preferences for behaviors and tailor the program accordingly to each individual.
Participants received a message with success of positive choices for behavioral action. The resulting actions and delivery of the intervention were automatically captured and downloaded in real time using iCardia and the Ilumivu mEMA smart phone app. Compliance to the EMI was considered <70% of time spent sedentary or a mean of > than 150 minutes of moderate physical activity/week as measured by the Fitbit (activity minutes). Participants randomized to the control group were sent and received an hour-long video education on general physical activity guidelines (questions were entertained and answered). At the completion of the 6-week intervention period all participants were again sent an ActiGraph to be worn on the waist for 7 days to measure sedentary time and daily physical activity. Once the ActiGraph was returned, a phone interview was scheduled with each participant to understand where they had difficulty and what aspects of the program did or did not work for them. The cognitive function tests administered at baseline were then repeated.

5-x) Clarify the level of human involvement

Clarify the level of human involvement (care providers or health professionals, also technical assistance) in the e-intervention or as co-intervention (detail number and expertise of professionals involved, if any, as well as “type of assistance offered, the timing and frequency of the support, how it is initiated, and the medium by which the assistance is delivered”. It may be necessary to distinguish between the level of human involvement required for the trial, and the level of human involvement required for a routine application outside of a RCT setting (discuss under item 21 – generalizability).

	1	2	3	4	5	
subitem not at all important	○	○	◉	○	○	essential

Clear selection

Does your paper address subitem 5-x?

Copy and paste relevant sections from the manuscript (include quotes in quotation marks "like this" to indicate direct quotes from your manuscript), or elaborate on this item by providing additional information not in the ms, or briefly explain why the item is not applicable/relevant for your study

The EMI program was developed based on the principles of the SEM, by understanding and considering participants' cultural and individual interaction with their living environment.37 We further individualized and adjusted the program based on participants stage of behavior change using the transtheoretical model using text messages designed in part with participant input that included health outcomes. The EMI program was also designed to provide motivational feedback of successes and encouragement via individualized text messages to empower participants in their own ability to achieve behavior change and gain positive results as explained by the social cognitive theory (SCT).38 We achieved this by interviewing each participant. Participants told investigators what times during the day they wanted the text message reminders and how the text messages should be phrased and what they preferred to do for physical activity based on their living-environment. They also provided their preference for which health benefit reminders to be sent and they helped design these to match each individual preference. Participants further told investigators what type of feedback they preferred and when they preferred the delivery of text message feedback of successes.

Participants in the EMI group were sent a Fitbit activity monitor and a research assistant helped them to set it up and configure it to receive sedentary behavior notifications in the form of vibrations when participant walked <250 steps per hour during waking hours (selected by the participant). We then scheduled a video call to set up a HIPPA compliant EMA system (Ilumivu mEMA) and to provide education about the Fitbit and the intervention to disrupt sedentary time. Through use of the Fitbit activity tracker, and the Ilumivu mEMA smart phone app, we were able to implement real-time delivery of the behavior options and feedback on the effectiveness of their behavior choices39 Although the intervention was guided by the individual interview findings, in general, participants received suggestions on their smartphone on how to replace sitting time with PA such as standing up 5 times or taking 20 steps, or even performing a short (20 seconds) preferred dance routine.

Participants received reminders from their smartphones to enter real-time feedback on activity options selected and how successful they were in adopting the option. These data allowed us to track underlying preferences for behaviors and tailor the program accordingly to each individual.

Participants received a message with success of positive choices for behavioral action. The resulting actions and delivery of the intervention were automatically captured and downloaded in real time using iCardia and the Ilumivu mEMA smart phone app. Compliance to the EMI was considered <70% of time spent sedentary or a mean of > than 150 minutes of moderate physical activity/week as measured by the Fitbit (activity minutes). Participants randomized to the control group were sent and received an hour-long video education on general physical activity guidelines (questions were entertained and answered). At the completion of the 6-week intervention period all participants were again sent an ActiGraph to be worn on the waist for 7 days to measure sedentary time and daily physical activity. Once the ActiGraph was returned, a phone interview was scheduled with each participant to understand where they had difficulty and what aspects of the program did or did not work for them. The cognitive function tests administered at baseline were then repeated.

5-xi) Report any prompts/reminders used

Report any prompts/reminders used: Clarify if there were prompts (letters, emails, phone calls, SMS) to use the application, what triggered them, frequency etc. It may be necessary to distinguish between the level of prompts/reminders required for the trial, and the level of prompts/reminders for a routine application outside of a RCT setting (discuss under item 21 – generalizability).

	1	2	3	4	5	
subitem not at all important	○	○	◉	○	○	essential

Clear selection

Does your paper address subitem 5-xi? *

Copy and paste relevant sections from the manuscript (include quotes in quotation marks "like this" to indicate direct quotes from your manuscript), or elaborate on this item by providing additional information not in the ms, or briefly explain why the item is not applicable/relevant for your study

The EMI program was developed based on the principles of the SEM, by understanding and considering participants' cultural and individual interaction with their living environment.37 We further individualized and adjusted the program based on participants stage of behavior change using the transtheoretical model using text messages designed in part with participant input that included health outcomes. The EMI program was also designed to provide motivational feedback of successes and encouragement via individualized text messages to empower participants in their own ability to achieve behavior change and gain positive results as explained by the social cognitive theory (SCT).38 We achieved this by interviewing each participant. Participants told investigators what times during the day they wanted the text message reminders and how the text messages should be phrased and what they preferred to do for physical activity based on their living-environment. They also provided their preference for which health benefit reminders to be sent and they helped design these to match each individual preference. Participants further told investigators what type of feedback they preferred and when they preferred the delivery of text message feedback of successes.

Participants in the EMI group were sent a Fitbit activity monitor and a research assistant helped them to set it up and configure it to receive sedentary behavior notifications in the form of vibrations when participant walked <250 steps per hour during waking hours (selected by the participant). We then scheduled a video call to set up a HIPPA compliant EMA system (Ilumivu mEMA) and to provide education about the Fitbit and the intervention to disrupt sedentary time. Through use of the Fitbit activity tracker, and the Ilumivu mEMA smart phone app, we were able to implement real-time delivery of the behavior options and feedback on the effectiveness of their behavior choices39 Although the intervention was guided by the individual interview findings, in general, participants received suggestions on their smartphone on how to replace sitting time with PA such as standing up 5 times or taking 20 steps, or even performing a short (20 seconds) preferred dance routine.

Participants received reminders from their smartphones to enter real-time feedback on activity options selected and how successful they were in adopting the option. These data allowed us to track underlying preferences for behaviors and tailor the program accordingly to each individual.

Participants received a message with success of positive choices for behavioral action. The resulting actions and delivery of the intervention were automatically captured and downloaded in real time using iCardia and the Ilumivu mEMA smart phone app. Compliance to the EMI was considered <70% of time spent sedentary or a mean of > than 150 minutes of moderate physical activity/week as measured by the Fitbit (activity minutes). Participants randomized to the control group were sent and received an hour-long video education on general physical activity guidelines (questions were entertained and answered). At the completion of the 6-week intervention period all participants were again sent an ActiGraph to be worn on the waist for 7 days to measure sedentary time and daily physical activity. Once the ActiGraph was returned, a phone interview was scheduled with each participant to understand where they had difficulty and what aspects of the program did or did not work for them. The cognitive function tests administered at baseline were then repeated.

5-xii) Describe any co-interventions (incl. training/support)

Describe any co-interventions (incl. training/support): Clearly state any interventions that are provided in addition to the targeted eHealth intervention, as ehealth intervention may not be designed as stand-alone intervention. This includes training sessions and support [1]. It may be necessary to distinguish between the level of training required for the trial, and the level of training for a routine application outside of a RCT setting (discuss under item 21 – generalizability.

	1	2	3	4	5	
subitem not at all important	○	○	◉	○	○	essential

Clear selection

Does your paper address subitem 5-xii? *

Copy and paste relevant sections from the manuscript (include quotes in quotation marks "like this" to indicate direct quotes from your manuscript), or elaborate on this item by providing additional information not in the ms, or briefly explain why the item is not applicable/relevant for your study

The EMI program was developed based on the principles of the SEM, by understanding and considering participants' cultural and individual interaction with their living environment.37 We further individualized and adjusted the program based on participants stage of behavior change using the transtheoretical model using text messages designed in part with participant input that included health outcomes. The EMI program was also designed to provide motivational feedback of successes and encouragement via individualized text messages to empower participants in their own ability to achieve behavior change and gain positive results as explained by the social cognitive theory (SCT).38 We achieved this by interviewing each participant. Participants told investigators what times during the day they wanted the text message reminders and how the text messages should be phrased and what they preferred to do for physical activity based on their living-environment. They also provided their preference for which health benefit reminders to be sent and they helped design these to match each individual preference. Participants further told investigators what type of feedback they preferred and when they preferred the delivery of text message feedback of successes.

Participants in the EMI group were sent a Fitbit activity monitor and a research assistant helped them to set it up and configure it to receive sedentary behavior notifications in the form of vibrations when participant walked <250 steps per hour during waking hours (selected by the participant). We then scheduled a video call to set up a HIPPA compliant EMA system (Ilumivu mEMA) and to provide education about the Fitbit and the intervention to disrupt sedentary time. Through use of the Fitbit activity tracker, and the Ilumivu mEMA smart phone app, we were able to implement real-time delivery of the behavior options and feedback on the effectiveness of their behavior choices39 Although the intervention was guided by the individual interview findings, in general, participants received suggestions on their smartphone on how to replace sitting time with PA such as standing up 5 times or taking 20 steps, or even performing a short (20 seconds) preferred dance routine.

Participants received reminders from their smartphones to enter real-time feedback on activity options selected and how successful they were in adopting the option. These data allowed us to track underlying preferences for behaviors and tailor the program accordingly to each individual.

Participants received a message with success of positive choices for behavioral action. The resulting actions and delivery of the intervention were automatically captured and downloaded in real time using iCardia and the Ilumivu mEMA smart phone app. Compliance to the EMI was considered <70% of time spent sedentary or a mean of > than 150 minutes of moderate physical activity/week as measured by the Fitbit (activity minutes). Participants randomized to the control group were sent and received an hour-long video education on general physical activity guidelines (questions were entertained and answered). At the completion of the 6-week intervention period all participants were again sent an ActiGraph to be worn on the waist for 7 days to measure sedentary time and daily physical activity. Once the ActiGraph was returned, a phone interview was scheduled with each participant to understand where they had difficulty and what aspects of the program did or did not work for them. The cognitive function tests administered at baseline were then repeated.

6a) Completely defined pre-specified primary and secondary outcome measures, including how and when they were assessed

Does your paper address CONSORT subitem 6a? *

Copy and paste relevant sections from the manuscript (include quotes in quotation marks "like this" to indicate direct quotes from your manuscript), or elaborate on this item by providing additional information not in the ms, or briefly explain why the item is not applicable/relevant for your study

Participants were first screened to determine eligibility via telephone and those that met criteria completed the informed e-consent conducted via Zoom video. Once consent was obtained, participants provided basic demographic information (age, sex, marital status, ethnicity, preferred language, and education) and a full medical history. We then completed the Charlson co-morbidity questionnaire for each participant and conducted the following NIH Toolbox cognitive function measures as approved by the NIH for use via video call:29 Picture vocabulary test, Picture sequence test, List sorting test, Oral reading recognition test, Auditory verbal learning test, and the Trail Making test A and B (TMT-A, and TMT-B)30-32. TMT-B time to completion was considered the measure of executive function.
Following cognitive function testing, we conducted a video interview, which was recorded, transcribed and translated, for the development of a culturally and individually tailored EMI program (see intervention). Once participants completed the interview portion, they were provided with a triaxial accelerometer (ActiGraph GT3X+, Actigraph, Pensacola, Florida), which they were instructed to wear on their right hip/waist for 7 consecutive days to measure sedentary time and daily physical activity. Upon return of the ActiGraph, participants were randomized and notified of their assigned group (EMI or physical activity guideline education). The accelerometer was worn by both groups at baseline and at follow up. Data collectors instructed the participants on the use do the accelerometer and the use of an accelerometer log.
Primary outcome measures:
The primary outcome measure was sedentary time, measured using the Actigraph. Sedentary time was defined as awake activity with an energy expenditure ≤ 1.5 metabolic equivalents occurring in a sitting or reclining posture; vector magnitude counts of <70 counts/15-s and vertical axis counts of <10 counts/15-s. Sedentary interruption was defined as counts >100/minute. Non-wear time was considered as 60 minutes of zero-activity counts. Any counts over 15,000 were considered erroneous33. A valid day consisted of 10 waking hours and at least 3 complete days of data (one person) had to be available to be included in the final analysis34. Data were processed with ActiLife version 6.13.5 software, with data converted to 60 seconds epochs. Non-wear time was defined as at least 60 consecutive minutes of 0 activity count. We categorized physical activity according to Freedson et al. (1998)35 cut-points.
Secondary outcome measures
The secondary outcome measures included cognitive function and feasibility and acceptability. Cognitive function was assessed using the auditory trail making test part A and part B, and the NIH Toolbox measures remote assessment.29 We captured Fitbit data remotely in real-time for compliance and fidelity of treatment to the EMI program using a digital health platform (iCardia)36. To analyze data obtained from the individual phone interviews, we conducted a thematic analysis to explore themes (manuscript in review). The thematic analysis provides a deeper understanding of physical, psychological, and social influences that influence physical activity choices in mid-life and older Latinos. Understanding these factors is crucial for continued refinement of effective interventions. Feasibility and acceptability measures were assessed by study satisfaction debriefing at the end of the study, a 10-point Likert scale, and using ecological momentary assessment during the course of the intervention period using a 10-point Likert scale. Feasibility was defined as a participant retention rate of >80%, a compliance rate to the intervention of >70% (defined as <70% of time spent sedentary or at least 150 minutes of moderate physical activity/week), and a wear time of activity tracking devices >80%. Acceptability was defined as 70% of participants rating >8/10 on study satisfaction Likert scale measures. All [illegible]

outcome measures were obtained at baseline and 6-weeks.

6a-i) Online questionnaires: describe if they were validated for online use and apply CHERRIES items to describe how the questionnaires were designed/deployed

If outcomes were obtained through online questionnaires, describe if they were validated for online use and apply CHERRIES items to describe how the questionnaires were designed/deployed [9].

	1	2	3	4	5	
subitem not at all important	○	○	◉	○	○	essential

Clear selection

Does your paper address subitem 6a-i?

Copy and paste relevant sections from manuscript text

N/A0-does not apply to this manuscript

6a-ii) Describe whether and how “use” (including intensity of use/dosage) was defined/measured/monitored

Describe whether and how “use” (including intensity of use/dosage) was defined/measured/monitored (logins, logfile analysis, etc.). Use/adoption metrics are important process outcomes that should be reported in any ehealth trial.

	1	2	3	4	5	
subitem not at all important	○	○	◉	○	○	essential

Clear selection

Does your paper address subitem 6a-ii?

Copy and paste relevant sections from manuscript text

Participants were first screened to determine eligibility via telephone and those that met criteria completed the informed e-consent conducted via Zoom video. Once consent was obtained, participants provided basic demographic information (age, sex, marital status, ethnicity, preferred language, and education) and a full medical history. We then completed the Charlson co-morbidity questionnaire for each participant and conducted the following NIH Toolbox cognitive function measures as approved by the NIH for use via video call:29 Picture vocabulary test, Picture sequence test, List sorting test, Oral reading recognition test, Auditory verbal learning test, and the Trail Making test A and B (TMT-A, and TMT-B)30-32. TMT-B time to completion was considered the measure of executive function.
Following cognitive function testing, we conducted a video interview, which was recorded, transcribed and translated, for the development of a culturally and individually tailored EMI program (see intervention). Once participants completed the interview portion, they were provided with a triaxial accelerometer (ActiGraph GT3X+, Actigraph, Pensacola, Florida), which they were instructed to wear on their right hip/waist for 7 consecutive days to measure sedentary time and daily physical activity. Upon return of the ActiGraph, participants were randomized and notified of their assigned group (EMI or physical activity guideline education). The accelerometer was worn by both groups at baseline and at follow up. Data collectors instructed the participants on the use do the accelerometer and the use of an accelerometer log.
Primary outcome measures:
The primary outcome measure was sedentary time, measured using the Actigraph. Sedentary time was defined as awake activity with an energy expenditure ≤ 1.5 metabolic equivalents occurring in a sitting or reclining posture; vector magnitude counts of <70 counts/15-s and vertical axis counts of <10 counts/15-s. Sedentary interruption was defined as counts >100/minute. Non-wear time was considered as 60 minutes of zero-activity counts. Any counts over 15,000 were considered erroneous33. A valid day consisted of 10 waking hours and at least 3 complete days of data (one person) had to be available to be included in the final analysis34. Data were processed with ActiLife version 6.13.5 software, with data converted to 60 seconds epochs. Non-wear time was defined as at least 60 consecutive minutes of 0 activity count. We categorized physical activity according to Freedson et al. (1998)35 cut-points.
Secondary outcome measures
The secondary outcome measures included cognitive function and feasibility and acceptability. Cognitive function was assessed using the auditory trail making test part A and part B, and the NIH Toolbox measures remote assessment.29 We captured Fitbit data remotely in real-time for compliance and fidelity of treatment to the EMI program using a digital health platform (iCardia)36. To analyze data obtained from the individual phone interviews, we conducted a thematic analysis to explore themes (manuscript in review). The thematic analysis provides a deeper understanding of physical, psychological, and social influences that influence physical activity choices in mid-life and older Latinos. Understanding these factors is crucial for continued refinement of effective interventions. Feasibility and acceptability measures were assessed by study satisfaction debriefing at the end of the study, a 10-point Likert scale, and using ecological momentary assessment during the course of the intervention period using a 10-point Likert scale. Feasibility was defined as a participant retention rate of >80%, a compliance rate to the intervention of >70% (defined as <70% of time spent sedentary or at least 150 minutes of moderate physical activity/week), and a wear time of activity tracking devices >80%. Acceptability was defined as 70% of participants rating >8/10 on study satisfaction Likert scale measures. All [illegible]

outcome measures were obtained at baseline and 6-weeks.

The EMI program was developed based on the principles of the SEM, by understanding and considering participants' cultural and individual interaction with their living environment.37 We further individualized and adjusted the program based on participants stage of behavior change using the transtheoretical model using text messages designed in part with participant input that included health outcomes. The EMI program was also designed to provide motivational feedback of successes and encouragement via individualized text messages to empower participants in their own ability to achieve behavior change and gain positive results as explained by the social cognitive theory (SCT).38 We achieved this by interviewing each participant. Participants told investigators what times during the day they wanted the text message reminders and how the text messages should be phrased and what they preferred to do for physical activity based on their living-environment. They also provided their preference for which health benefit reminders to be sent and they helped design these to match each individual preference. Participants further told investigators what type of feedback they preferred and when they preferred the delivery of text message feedback of successes.

Participants in the EMI group were sent a Fitbit activity monitor and a research assistant helped them to set it up and configure it to receive sedentary behavior notifications in the form of vibrations when participant walked <250 steps per hour during waking hours (selected by the participant). We then scheduled a video call to set up a HIPPA compliant EMA system (Ilumivu mEMA) and to provide education about the Fitbit and the intervention to disrupt sedentary time. Through use of the Fitbit activity tracker, and the Ilumivu mEMA smart phone app, we were able to implement real-time delivery of the behavior options and feedback on the effectiveness of their behavior choices39 Although the intervention was guided by the individual interview findings, in general, participants received suggestions on their smartphone on how to replace sitting time with PA such as standing up 5 times or taking 20 steps, or even performing a short (20 seconds) preferred dance routine.

Participants received reminders from their smartphones to enter real-time feedback on activity options selected and how successful they were in adopting the option. These data allowed us to track underlying preferences for behaviors and tailor the program accordingly to each individual.

Participants received a message with success of positive choices for behavioral action. The resulting actions and delivery of the intervention were automatically captured and downloaded in real time using iCardia and the Ilumivu mEMA smart phone app. Compliance to the EMI was considered <70% of time spent sedentary or a mean of > than 150 minutes of moderate physical activity/week as measured by the Fitbit (activity minutes). Participants randomized to the control group were sent and received an hour-long video education on general physical activity guidelines (questions were entertained and answered). At the completion of the 6-week intervention period all participants were again sent an ActiGraph to be worn on the waist for 7 days to measure sedentary time and daily physical activity. Once the ActiGraph was returned, a phone interview was scheduled with each participant to understand where they had difficulty and what aspects of the program did or did not work for them. The cognitive function tests administered at baseline were then repeated.

6a-iii) Describe whether, how, and when qualitative feedback from participants was obtained

Describe whether, how, and when qualitative feedback from participants was obtained (e.g., through emails, feedback forms, interviews, focus groups).

	1	2	3	4	5	
subitem not at all important	○	○	◉	○	○	essential

Clear selection

Does your paper address subitem 6a-iii?

Copy and paste relevant sections from manuscript text

The EMI program was developed based on the principles of the SEM, by understanding and considering participants' cultural and individual interaction with their living environment.37 We further individualized and adjusted the program based on participants stage of behavior change using the transtheoretical model using text messages designed in part with participant input that included health outcomes. The EMI program was also designed to provide motivational feedback of successes and encouragement via individualized text messages to empower participants in their own ability to achieve behavior change and gain positive results as explained by the social cognitive theory (SCT).38 We achieved this by interviewing each participant. Participants told investigators what times during the day they wanted the text message reminders and how the text messages should be phrased and what they preferred to do for physical activity based on their living-environment. They also provided their preference for which health benefit reminders to be sent and they helped design these to match each individual preference. Participants further told investigators what type of feedback they preferred and when they preferred the delivery of text message feedback of successes.

Participants in the EMI group were sent a Fitbit activity monitor and a research assistant helped them to set it up and configure it to receive sedentary behavior notifications in the form of vibrations when participant walked <250 steps per hour during waking hours (selected by the participant). We then scheduled a video call to set up a HIPPA compliant EMA system (Ilumivu mEMA) and to provide education about the Fitbit and the intervention to disrupt sedentary time. Through use of the Fitbit activity tracker, and the Ilumivu mEMA smart phone app, we were able to implement real-time delivery of the behavior options and feedback on the effectiveness of their behavior choices39 Although the intervention was guided by the individual interview findings, in general, participants received suggestions on their smartphone on how to replace sitting time with PA such as standing up 5 times or taking 20 steps, or even performing a short (20 seconds) preferred dance routine.

Participants received reminders from their smartphones to enter real-time feedback on activity options selected and how successful they were in adopting the option. These data allowed us to track underlying preferences for behaviors and tailor the program accordingly to each individual.

Participants received a message with success of positive choices for behavioral action. The resulting actions and delivery of the intervention were automatically captured and downloaded in real time using iCardia and the Ilumivu mEMA smart phone app. Compliance to the EMI was considered <70% of time spent sedentary or a mean of > than 150 minutes of moderate physical activity/week as measured by the Fitbit (activity minutes). Participants randomized to the control group were sent and received an hour-long video education on general physical activity guidelines (questions were entertained and answered). At the completion of the 6-week intervention period all participants were again sent an ActiGraph to be worn on the waist for 7 days to measure sedentary time and daily physical activity. Once the ActiGraph was returned, a phone interview was scheduled with each participant to understand where they had difficulty and what aspects of the program did or did not work for them. The cognitive function tests administered at baseline were then repeated.

6b) Any changes to trial outcomes after the trial commenced, with reasons

Does your paper address CONSORT subitem 6b? *

Copy and paste relevant sections from the manuscript (include quotes in quotation marks "like this" to indicate direct quotes from your manuscript), or elaborate on this item by providing additional information not in the ms, or briefly explain why the item is not applicable/relevant for your study

No changes were made to this outcomes after the trial began

7a) How sample size was determined

NPT: When applicable, details of whether and how the clustering by care provides or centers was addressed

7a-i) Describe whether and how expected attrition was taken into account when calculating the sample size

Describe whether and how expected attrition was taken into account when calculating the sample size.

	1	2	3	4	5	
subitem not at all important	○	○	◉	○	○	essential

Clear selection

Does your paper address subitem 7a-i?

Copy and paste relevant sections from manuscript title (include quotes in quotation marks "like this" to indicate direct quotes from your manuscript), or elaborate on this item by providing additional information not in the ms, or briefly explain why the item is not applicable/relevant for your study

This was a pilot trial and therefore no power calculation was conducted. This was a pilot randomized controlled trial for feasibility, acceptability, and effect. Therefore, we emphasized descriptive statistics such as means, standard deviations, frequencies, percentages, and effect sizes, to demonstrate the feasibility of recruitment, adherence, retention, treatment effects over time, and proof of concept.

7b) When applicable, explanation of any interim analyses and stopping guidelines

Does your paper address CONSORT subitem 7b? *

Copy and paste relevant sections from the manuscript (include quotes in quotation marks "like this" to indicate direct quotes from your manuscript), or elaborate on this item by providing additional information not in the ms, or briefly explain why the item is not applicable/relevant for your study

No interim analysis or stopping guidelines were required.

8a) Method used to generate the random allocation sequence

NPT: When applicable, how care providers were allocated to each trial group

Does your paper address CONSORT subitem 8a? *

Copy and paste relevant sections from the manuscript (include quotes in quotation marks "like this" to indicate direct quotes from your manuscript), or elaborate on this item by providing additional information not in the ms, or briefly explain why the item is not applicable/relevant for your study

Participants were randomized using computerized variable block randomization of two and four as designed by the project statistician not involved in recruitment.

8b) Type of randomisation; details of any restriction (such as blocking and block size)

Does your paper address CONSORT subitem 8b? *

Copy and paste relevant sections from the manuscript (include quotes in quotation marks "like this" to indicate direct quotes from your manuscript), or elaborate on this item by providing additional information not in the ms, or briefly explain why the item is not applicable/relevant for your study

Participants were randomized using computerized variable block randomization of two and four as designed by the project statistician not involved in recruitment.

9) Mechanism used to implement the random allocation sequence (such as sequentially numbered containers), describing any steps taken to conceal the sequence until interventions were assigned

Does your paper address CONSORT subitem 9? *

Copy and paste relevant sections from the manuscript (include quotes in quotation marks "like this" to indicate direct quotes from your manuscript), or elaborate on this item by providing additional information not in the ms, or briefly explain why the item is not applicable/relevant for your study

Participants were randomized using computerized variable block randomization of two and four as designed by the project statistician not involved in recruitment.

10) Who generated the random allocation sequence, who enrolled participants, and who assigned participants to interventions

Does your paper address CONSORT subitem 10? *

Copy and paste relevant sections from the manuscript (include quotes in quotation marks "like this" to indicate direct quotes from your manuscript), or elaborate on this item by providing additional information not in the ms, or briefly explain why the item is not applicable/relevant for your study

Participants were randomized using computerized variable block randomization of two and four as designed by the project statistician not involved in recruitment.

11a) If done, who was blinded after assignment to interventions (for example, participants, care providers, those assessing outcomes) and how

NPT: Whether or not administering co-interventions were blinded to group assignment

11a-i) Specify who was blinded, and who wasn't

Specify who was blinded, and who wasn't. Usually, in web-based trials it is not possible to blind the participants [1, 3] (this should be clearly acknowledged), but it may be possible to blind outcome assessors, those doing data analysis or those administering co-interventions (if any).

	1	2	3	4	5	
subitem not at all important	○	○	●	○	○	essential

Clear selection

Does your paper address subitem 11a-i? *

Copy and paste relevant sections from the manuscript (include quotes in quotation marks "like this" to indicate direct quotes from your manuscript), or elaborate on this item by providing additional information not in the ms, or briefly explain why the item is not applicable/relevant for your study

The PI was blinded and we used alternate forms for assessment as baseline and follow up

11a-ii) Discuss e.g., whether participants knew which intervention was the “intervention of interest” and which one was the “comparator”

Informed consent procedures (4a-ii) can create biases and certain expectations - discuss e.g., whether participants knew which intervention was the “intervention of interest” and which one was the “comparator”.

	1	2	3	4	5	
subitem not at all important	○	○	◉	○	○	essential

Clear selection

Does your paper address subitem 11a-ii?

Copy and paste relevant sections from the manuscript (include quotes in quotation marks "like this" to indicate direct quotes from your manuscript), or elaborate on this item by providing additional information not in the ms, or briefly explain why the item is not applicable/relevant for your study

Not applicable. This was a physical activity trial and it is not possible to conceal the intervention.

11b) If relevant, description of the similarity of interventions

(this item is usually not relevant for ehealth trials as it refers to similarity of a placebo or sham intervention to a active medication/intervention)

Does your paper address CONSORT subitem 11b? *

Copy and paste relevant sections from the manuscript (include quotes in quotation marks "like this" to indicate direct quotes from your manuscript), or elaborate on this item by providing additional information not in the ms, or briefly explain why the item is not applicable/relevant for your study

Not relevant. Control group received physical activity guideline education.

12a) Statistical methods used to compare groups for primary and secondary outcomes

NPT: When applicable, details of whether and how the clustering by care providers or centers was addressed

Does your paper address CONSORT subitem 12a? *

Copy and paste relevant sections from the manuscript (include quotes in quotation marks "like this" to indicate direct quotes from your manuscript), or elaborate on this item by providing additional information not in the ms, or briefly explain why the item is not applicable/relevant for your study

This was a pilot randomized controlled trial for feasibility, acceptability, and effect. Therefore, we emphasized descriptive statistics such as means, standard deviations, frequencies, percentages, and effect sizes, to demonstrate the feasibility of recruitment, adherence, retention, treatment effects over time, and proof of concept. Two-sample t-tests and chi-square tests (using Monte Carlo simulation to obtain p-values) were used to test for baseline demographic differences across randomized groups. When evaluating primary and secondary outcomes, paired t-tests were used to assess within group change from baseline to six-weeks. Cohen’s D was used to estimate the effect of the intervention (change in EMI vs. control). Linear models predicting change scores by treatment group (unadjusted model), and similar models adjusting for Age, sex and years of education were computed for each outcome measure. P<0.05 with two-sided tests were used to assess statistical significance, though p<0.20 was considered a trend towards significance given the pilot nature of the study.

12a-i) Imputation techniques to deal with attrition / missing values

Imputation techniques to deal with attrition / missing values: Not all participants will use the intervention/comparator as intended and attrition is typically high in ehealth trials. Specify how participants who did not use the application or dropped out from the trial were treated in the statistical analysis (a complete case analysis is strongly discouraged, and simple imputation techniques such as LOCF may also be problematic [4]).

	1	2	3	4	5	
subitem not at all important	○	○	●	○	○	essential

Clear selection

Does your paper address subitem 12a-i? *

Copy and paste relevant sections from the manuscript (include quotes in quotation marks "like this" to indicate direct quotes from your manuscript), or elaborate on this item by providing additional information not in the ms, or briefly explain why the item is not applicable/relevant for your study

There was no missing data for this study.

12b) Methods for additional analyses, such as subgroup analyses and adjusted analyses

Does your paper address CONSORT subitem 12b? *

Copy and paste relevant sections from the manuscript (include quotes in quotation marks "like this" to indicate direct quotes from your manuscript), or elaborate on this item by providing additional information not in the ms, or briefly explain why the item is not applicable/relevant for your study

This was a pilot randomized controlled trial for feasibility, acceptability, and effect. Therefore, we emphasized descriptive statistics such as means, standard deviations, frequencies, percentages, and effect sizes, to demonstrate the feasibility of recruitment, adherence, retention, treatment effects over time, and proof of concept. Two-sample t-tests and chi-square tests (using Monte Carlo simulation to obtain p-values) were used to test for baseline demographic differences across randomized groups. When evaluating primary and secondary outcomes, paired t-tests were used to assess within group change from baseline to six-weeks. Cohen's D was used to estimate the effect of the intervention (change in EMI vs. control). Linear models predicting change scores by treatment group (unadjusted model), and similar models adjusting for Age, sex and years of education were computed for each outcome measure. $P<0.05$ with two-sided tests were used to assess statistical significance, though $p<0.20$ was considered a trend towards significance given the pilot nature of the study.

X26) REB/IRB Approval and Ethical Considerations [recommended as subheading under "Methods"] (not a CONSORT item)

X26-i) Comment on ethics committee approval

	1	2	3	4	5	
subitem not at all important	○	○	●	○	○	essential

Clear selection

Does your paper address subitem X26-i?

Copy and paste relevant sections from the manuscript (include quotes in quotation marks "like this" to indicate direct quotes from your manuscript), or elaborate on this item by providing additional information not in the ms, or briefly explain why the item is not applicable/relevant for your study

The study was approved by the University of Illinois at Chicago (UIC) Institutional Review Board (2020-0739) and informed consent was obtained prior to any study procedures.

x26-ii) Outline informed consent procedures

Outline informed consent procedures e.g., if consent was obtained offline or online (how? Checkbox, etc.?), and what information was provided (see 4a-ii). See [6] for some items to be included in informed consent documents.

	1	2	3	4	5	
subitem not at all important	○	○	●	○	○	essential

Clear selection

Does your paper address subitem X26-ii?

Copy and paste relevant sections from the manuscript (include quotes in quotation marks "like this" to indicate direct quotes from your manuscript), or elaborate on this item by providing additional information not in the ms, or briefly explain why the item is not applicable/relevant for your study

Participants were first screened to determine eligibility via telephone and those that met criteria completed the informed e-consent conducted via Zoom video. Once consent was obtained, participants provided basic demographic information (age, sex, marital status, ethnicity, preferred language, and education) and a full medical history.

X26-iii) Safety and security procedures

Safety and security procedures, incl. privacy considerations, and any steps taken to reduce the likelihood or detection of harm (e.g., education and training, availability of a hotline)

	1	2	3	4	5	
subitem not at all important	○	○	◉	○	○	essential

Clear selection

Does your paper address subitem X26-iii?

Copy and paste relevant sections from the manuscript (include quotes in quotation marks "like this" to indicate direct quotes from your manuscript), or elaborate on this item by providing additional information not in the ms, or briefly explain why the item is not applicable/relevant for your study

Your answer

RESULTS

13a) For each group, the numbers of participants who were randomly assigned, received intended treatment, and were analysed for the primary outcome

NPT: The number of care providers or centers performing the intervention in each group and the number of patients treated by each care provider in each center

Does your paper address CONSORT subitem 13a? *

Copy and paste relevant sections from the manuscript (include quotes in quotation marks "like this" to indicate direct quotes from your manuscript), or elaborate on this item by providing additional information not in the ms, or briefly explain why the item is not applicable/relevant for your study

This is listed in the consort diagram. Of the 69 participants assessed for eligibility, 19 did not meet inclusion criteria, and 50 participants met inclusion criteria. Of the 50 participants that met inclusion criteria,11 participants declined participation due to disinterest/time constraints. Thus, 39 participants were randomized to either the ecological momentary intervention (EMI; N=20) or a group receiving education on physical activity guidelines (control; N=19) (1). One participant was lost to follow up after randomization, but prior to intervention initiation due to leaving the country permanently (CONSORT flow diagram, Figure 1).

13b) For each group, losses and exclusions after randomisation, together with reasons

Does your paper address CONSORT subitem 13b? (NOTE: Preferably, this is shown in a CONSORT flow diagram) *

Copy and paste relevant sections from the manuscript (include quotes in quotation marks "like this" to indicate direct quotes from your manuscript), or elaborate on this item by providing additional information not in the ms, or briefly explain why the item is not applicable/relevant for your study

Of the 69 participants assessed for eligibility, 19 did not meet inclusion criteria, and 50 participants met inclusion criteria. Of the 50 participants that met inclusion criteria,11 participants declined participation due to disinterest/time constraints. Thus, 39 participants were randomized to either the ecological momentary intervention (EMI; N=20) or a group receiving education on physical activity guidelines (control; N=19) (1). One participant was lost to follow up after randomization, but prior to intervention initiation due to leaving the country permanently (CONSORT flow diagram, Figure 1).

13b-i) Attrition diagram

Strongly recommended: An attrition diagram (e.g., proportion of participants still logging in or using the intervention/comparator in each group plotted over time, similar to a survival curve) or other figures or tables demonstrating usage/dose/engagement.

	1	2	3	4	5	
subitem not at all important	○	○	◉	○	○	essential

Clear selection

Does your paper address subitem 13b-i?

Copy and paste relevant sections from the manuscript or cite the figure number if applicable (include quotes in quotation marks "like this" to indicate direct quotes from your manuscript), or elaborate on this item by providing additional information not in the ms, or briefly explain why the item is not applicable/relevant for your study

Of the 69 participants assessed for eligibility, 19 did not meet inclusion criteria, and 50 participants met inclusion criteria. Of the 50 participants that met inclusion criteria,11 participants declined participation due to disinterest/time constraints. Thus, 39 participants were randomized to either the ecological momentary intervention (EMI; N=20) or a group receiving education on physical activity guidelines (control; N=19) (1). One participant was lost to follow up after randomization, but prior to intervention initiation due to leaving the country permanently (CONSORT flow diagram, Figure 1).

14a) Dates defining the periods of recruitment and follow-up

Does your paper address CONSORT subitem 14a? *

Copy and paste relevant sections from the manuscript (include quotes in quotation marks "like this" to indicate direct quotes from your manuscript), or elaborate on this item by providing additional information not in the ms, or briefly explain why the item is not applicable/relevant for your study

This was a 6-week study.

14a-i) Indicate if critical "secular events" fell into the study period

Indicate if critical "secular events" fell into the study period, e.g., significant changes in Internet resources available or "changes in computer hardware or Internet delivery resources"

	1	2	3	4	5	
subitem not at all important	○	○	◉	○	○	essential

Clear selection

Does your paper address subitem 14a-i?

Copy and paste relevant sections from the manuscript (include quotes in quotation marks "like this" to indicate direct quotes from your manuscript), or elaborate on this item by providing additional information not in the ms, or briefly explain why the item is not applicable/relevant for your study

This is not applicable to this study. No changes were made

14b) Why the trial ended or was stopped (early)

Does your paper address CONSORT subitem 14b? *

Copy and paste relevant sections from the manuscript (include quotes in quotation marks "like this" to indicate direct quotes from your manuscript), or elaborate on this item by providing additional information not in the ms, or briefly explain why the item is not applicable/relevant for your study

No. This trial was not stopped early.

15) A table showing baseline demographic and clinical characteristics for each group

NPT: When applicable, a description of care providers (case volume, qualification, expertise, etc.) and centers (volume) in each group

Does your paper address CONSORT subitem 15? *

Copy and paste relevant sections from the manuscript (include quotes in quotation marks "like this" to indicate direct quotes from your manuscript), or elaborate on this item by providing additional information not in the ms, or briefly explain why the item is not applicable/relevant for your study

Table one shows this. The two randomized groups did not show evidence of difference in baseline demographics, medication use and medical history, except for the EMI group consisting of slightly more individuals indicating they were married (Table 1). The majority of the sample (95%; 37/39) identified as Latino only with only two participants identifying as Hispanic/Latino-White (5%=2/39). The sample was representative of the Chicago Latino community in terms of education albeit slightly below those published in the Chicago 2022 Community Survey.40 When compared with the Chicago 2022 Latino Community Survey,40 32% vs. 29% had < a high school education; 34% vs. 28% had a high school education; and 15.8%= vs. 20 % had an Associate's or bachelor's degree.

15-i) Report demographics associated with digital divide issues

In ehealth trials it is particularly important to report demographics associated with digital divide issues, such as age, education, gender, social-economic status, computer/Internet/ehealth literacy of the participants, if known.

	1	2	3	4	5	
subitem not at all important	○	○	●	○	○	essential

Clear selection

Does your paper address subitem 15-i? *

Copy and paste relevant sections from the manuscript (include quotes in quotation marks "like this" to indicate direct quotes from your manuscript), or elaborate on this item by providing additional information not in the ms, or briefly explain why the item is not applicable/relevant for your study

The two randomized groups did not show evidence of difference in baseline demographics, medication use and medical history, except for the EMI group consisting of slightly more individuals indicating they were married (Table 1). The majority of the sample (95%; 37/39) identified as Latino only with only two participants identifying as Hispanic/Latino-White (5%=2/39). The sample was representative of the Chicago Latino community in terms of education albeit slightly below those published in the Chicago 2022 Community Survey.40 When compared with the Chicago 2022 Latino Community Survey,40 32% vs. 29% had < a high school education; 34% vs. 28% had a high school education; and 15.8%= vs. 20 % had an Associate's or bachelor's degree.

16) For each group, number of participants (denominator) included in each analysis and whether the analysis was by original assigned groups

16-i) Report multiple "denominators" and provide definitions

Report multiple "denominators" and provide definitions: Report N's (and effect sizes) "across a range of study participation [and use] thresholds" [1], e.g., N exposed, N consented, N used more than x times, N used more than y weeks, N participants "used" the intervention/comparator at specific pre-defined time points of interest (in absolute and relative numbers per group). Always clearly define "use" of the intervention.

	1	2	3	4	5	
subitem not at all important	○	○	●	○	○	essential

Clear selection

Does your paper address subitem 16-i? *

Copy and paste relevant sections from the manuscript (include quotes in quotation marks "like this" to indicate direct quotes from your manuscript), or elaborate on this item by providing additional information not in the ms, or briefly explain why the item is not applicable/relevant for your study

This is addressed in the consort diagram. 19 participants in the intervention and 19 participants in the control for all outcome assessments.

16-ii) Primary analysis should be intent-to-treat

Primary analysis should be intent-to-treat, secondary analyses could include comparing only “users”, with the appropriate caveats that this is no longer a randomized sample (see 18-i).

	1	2	3	4	5	
subitem not at all important	○	○	●	○	○	essential

Clear selection

Does your paper address subitem 16-ii?

Copy and paste relevant sections from the manuscript (include quotes in quotation marks "like this" to indicate direct quotes from your manuscript), or elaborate on this item by providing additional information not in the ms, or briefly explain why the item is not applicable/relevant for your study

Intention to treat was not conducted due to this being a pilot study. We had only one subject that dropped out. This subject dropped out prior to intervention taking place and left the country. We elected to conducted analyses with 19 subjects in each group. This was a pilot randomized controlled trial for feasibility, acceptability, and effect. Therefore, we emphasized descriptive statistics such as means, standard deviations, frequencies, percentages, and effect sizes, to demonstrate the feasibility of recruitment, adherence, retention, treatment effects over time, and proof of concept. Two-sample t-tests and chi-square tests (using Monte Carlo simulation to obtain p-values) were used to test for baseline demographic differences across randomized groups. When evaluating primary and secondary outcomes, paired t-tests were used to assess within group change from baseline to six-weeks. Cohen's D was used to estimate the effect of the intervention (change in EMI vs. control). Linear models predicting change scores by treatment group (unadjusted model), and similar models adjusting for Age, sex and years of education were computed for each outcome measure. P<0.05 with two-sided tests were used to assess statistical significance, though p<0.20 was considered a trend towards significance given the pilot nature of the study.

17a) For each primary and secondary outcome, results for each group, and the estimated effect size and its precision (such as 95% confidence interval)

Does your paper address CONSORT subitem 17a? *

Copy and paste relevant sections from the manuscript (include quotes in quotation marks "like this" to indicate direct quotes from your manuscript), or elaborate on this item by providing additional information not in the ms, or briefly explain why the item is not applicable/relevant for your study

We provide these values in the tables of results.
Primary outcome: Sedentary time
A variety of measures of sedentary time were obtained from Actigraph data (Table 2). Across all twelve measures, the EMI group showed greater improvement in sedentary time as compared to the control group (fewer sedentary bouts, more sedentary breaks, and less sedentary time overall). Five of the 12 measures showed statistically significant improvement ($p<0.05$) in the EMI group from baseline, with an additional four measures showing a positive trend ($p<0.12$). Evidence of change was much less in the control group ($p>0.5$ for 9 of 12 measures, and $p>0.13$ in all cases). Overall effect size estimates (Cohen's d) comparing the two treatments were >0.3 for 9 of 12 measures and >0.05 in all 12 cases, however these unadjusted (and adjusted) effects did not attain statistical significance ($p>0.10$ in all cases). A variety of measures of daily physical activity were obtained from Actigraph data (Table 3). Across all 11 measures, the EMI group showed greater improvement in activity as compared to the control group (light, moderate and vigorous activity minutes/day as well as percent of daily activities). Three of the 11 measures showed statistically significant improvement ($p<0.05$) in the EMI group from baseline, with an additional three measures showing a positive trend ($p<0.15$). Evidence of change was much less in the control group ($p\geq0.4$ for all 11 measures). Overall effect size estimates (Cohen's d) comparing the two treatments were ≥0.3 for 6 of 11 measures (with four of the remaining measures having $d>0$), however these unadjusted (and adjusted) effects did not attain statistical significance in most cases ($p<0.05$ in one unadjusted and three adjusted models).
Secondary outcome: Cognitive Performance
Cognitive performance was assessed with the Trail making test part A and B as well as the NIH toolbox, with 9 total assessed measures (Table 4). Across all 11 measures, the EMI group showed greater improvement in cognitive performance as compared to the control group. One of the 9 measures showed statistically significant improvement ($p<0.05$) in the EMI group from baseline, with an additional four measures showing a positive trend ($p<0.20$). Evidence of change was much less in the control group ($p\geq0.4$ for 8 of 9 measures). Overall effect size estimates (Cohen's d) comparing the two treatments were ≥0.3 for 5 of 9 measures (with the 4 remaining measures having $d>0$), however these unadjusted (and adjusted) effects did not attain statistical significance in most cases ($p<0.05$ in one unadjusted and two adjusted models).

17a-i) Presentation of process outcomes such as metrics of use and intensity of use

In addition to primary/secondary (clinical) outcomes, the presentation of process outcomes such as metrics of use and intensity of use (dose, exposure) and their operational definitions is critical. This does not only refer to metrics of attrition (13-b) (often a binary variable), but also to more continuous exposure metrics such as “average session length”. These must be accompanied by a technical description how a metric like a “session” is defined (e.g., timeout after idle time) [1] (report under item 6a).

	1	2	3	4	5	
subitem not at all important	○	○	●	○	○	essential

Clear selection

Does your paper address subitem 17a-i?

Copy and paste relevant sections from the manuscript (include quotes in quotation marks "like this" to indicate direct quotes from your manuscript), or elaborate on this item by providing additional information not in the ms, or briefly explain why the item is not applicable/relevant for your study

Baseline interviews provided valuable insight into the perceived barriers, motivators, and facilitators to breaking up sedentary behavior as reported by participants. Environmental and social factors such as sedentary working conditions, weather, and social distancing during the COVID-19 pandemic, were cited as major barriers to physical activity. Interestingly, the major motivators and facilitators reported by participants included both internal and external sources. Self-motivation to maintain good health and general awareness that poor health will lead to increased sedentary behavior was reported. Social networks and the support garnered from them were cited as highly influential to one's motivation to remain physically active throughout their day. A complete description of the interview findings is beyond the scope of this manuscript and will be published elsewhere (manuscript in review). Compliance to the EMI program was 79% defined as <70% awake time spent sedentary or 150 minutes of moderate activity/week, study satisfaction was high, and the mean minutes of activity minutes per week was 382 minutes/week captured via Fibit. The participants expressed a high degree of acceptability (Table 4). A total of 2,850 text messages were sent out. All of them were successfully delivered (100% rate). EMA survey results from 1,394 survey messages over 6-weeks showed that EMI participants found the text message suggestions to move to be highly motivating 82.4% of the time (8-10 on a 0-10 Likert scale). EMI participant enjoyed the text messages 84.4%, and participants found that the Fitbit was motivating in conjunction with receiving text message suggestions to move 90.9% of the time, while 92.7% found little-to-no difficulty using the Fitbit tracker and mobile app (0-2 on a 0-10 Likert scale).

17b) For binary outcomes, presentation of both absolute and relative effect sizes is recommended

Does your paper address CONSORT subitem 17b? *

Copy and paste relevant sections from the manuscript (include quotes in quotation marks "like this" to indicate direct quotes from your manuscript), or elaborate on this item by providing additional information not in the ms, or briefly explain why the item is not applicable/relevant for your study

This was not conducted in this study

18) Results of any other analyses performed, including subgroup analyses and adjusted analyses, distinguishing pre-specified from exploratory

Does your paper address CONSORT subitem 18? *

Copy and paste relevant sections from the manuscript (include quotes in quotation marks "like this" to indicate direct quotes from your manuscript), or elaborate on this item by providing additional information not in the ms, or briefly explain why the item is not applicable/relevant for your study

Your answer

18-i) Subgroup analysis of comparing only users

A subgroup analysis of comparing only users is not uncommon in ehealth trials, but if done, it must be stressed that this is a self-selected sample and no longer an unbiased sample from a randomized trial (see 16-iii).

	1	2	3	4	5	
subitem not at all important	○	○	◉	○	○	essential

Clear selection

Does your paper address subitem 18-i?

Copy and paste relevant sections from the manuscript (include quotes in quotation marks "like this" to indicate direct quotes from your manuscript), or elaborate on this item by providing additional information not in the ms, or briefly explain why the item is not applicable/relevant for your study

Exploratory mediation analysis
In unadjusted models the association of intervention and change in TMT-B was 42.46 seconds (p=0.02), and the association of intervention and change in percent MVPA was 1.04 (p=0.038). The association of change in TMT-B and change in percent MVPA was -17.47, indicating that 1 percent increase in exercise was associated with a 17.47 second increase in TMT-B (p=0.003).
After accounting for change in exercise the association of intervention and change in Trails B was 27.42 seconds (p=0.12). This exploratory analysis suggests that approximately 35% of the impact of the intervention on Trails B is accounted for by changes in percent MVPA.

19) All important harms or unintended effects in each group
(for specific guidance see CONSORT for harms)

Does your paper address CONSORT subitem 19? *

Copy and paste relevant sections from the manuscript (include quotes in quotation marks "like this" to indicate direct quotes from your manuscript), or elaborate on this item by providing additional information not in the ms, or briefly explain why the item is not applicable/relevant for your study

This is addressed in the CONSORT diagram

19-i) Include privacy breaches, technical problems

Include privacy breaches, technical problems. This does not only include physical “harm” to participants, but also incidents such as perceived or real privacy breaches [1], technical problems, and other unexpected/unintended incidents. “Unintended effects” also includes unintended positive effects [2].

	1	2	3	4	5	
subitem not at all important	○	○	◉	○	○	essential

Clear selection

Does your paper address subitem 19-i?

Copy and paste relevant sections from the manuscript (include quotes in quotation marks "like this" to indicate direct quotes from your manuscript), or elaborate on this item by providing additional information not in the ms, or briefly explain why the item is not applicable/relevant for your study

We did not have any breaches or technical problems.

19-ii) Include qualitative feedback from participants or observations from staff/researchers

Include qualitative feedback from participants or observations from staff/researchers, if available, on strengths and shortcomings of the application, especially if they point to unintended/unexpected effects or uses. This includes (if available) reasons for why people did or did not use the application as intended by the developers.

	1	2	3	4	5	
subitem not at all important	○	○	◉	○	○	essential

Clear selection

Does your paper address subitem 19-ii?

Copy and paste relevant sections from the manuscript (include quotes in quotation marks "like this" to indicate direct quotes from your manuscript), or elaborate on this item by providing additional information not in the ms, or briefly explain why the item is not applicable/relevant for your study

Baseline interviews provided valuable insight into the perceived barriers, motivators, and facilitators to breaking up sedentary behavior as reported by participants. Environmental and social factors such as sedentary working conditions, weather, and social distancing during the COVID-19 pandemic, were cited as major barriers to physical activity. Interestingly, the major motivators and facilitators reported by participants included both internal and external sources. Self-motivation to maintain good health and general awareness that poor health will lead to increased sedentary behavior was reported. Social networks and the support garnered from them were cited as highly influential to one's motivation to remain physically active throughout their day. A complete description of the interview findings is beyond the scope of this manuscript and will be published elsewhere (manuscript in review). Compliance to the EMI program was 79% defined as <70% awake time spent sedentary or 150 minutes of moderate activity/week, study satisfaction was high, and the mean minutes of activity minutes per week was 382 minutes/week captured via Fibit. The participants expressed a high degree of acceptability (Table 4). A total of 2,850 text messages were sent out. All of them were successfully delivered (100% rate). EMA survey results from 1,394 survey messages over 6-weeks showed that EMI participants found the text message suggestions to move to be highly motivating 82.4% of the time (8-10 on a 0-10 Likert scale). EMI participant enjoyed the text messages 84.4%, and participants found that the Fitbit was motivating in conjunction with receiving text message suggestions to move 90.9% of the time, while 92.7% found little-to-no difficulty using the Fitbit tracker and mobile app (0-2 on a 0-10 Likert scale).

DISCUSSION

22) Interpretation consistent with results, balancing benefits and harms, and considering other relevant evidence

NPT: In addition, take into account the choice of the comparator, lack of or partial blinding, and unequal expertise of care providers or centers in each group

22-i) Restate study questions and summarize the answers suggested by the data, starting with primary outcomes and process outcomes (use)

Restate study questions and summarize the answers suggested by the data, starting with primary outcomes and process outcomes (use).

	1	2	3	4	5	
subitem not at all important	○	○	◉	○	○	essential

Clear selection

Does your paper address subitem 22-i? *

Copy and paste relevant sections from the manuscript (include quotes in quotation marks "like this" to indicate direct quotes from your manuscript), or elaborate on this item by providing additional information not in the ms, or briefly explain why the item is not applicable/relevant for your study

This study investigated the impact of a culturally appropriate, individualized, ecological momentary intervention program on breaking up and replacing sedentary time (sitting) with physical activity on cognitive performance in older Latinos with Spanish speaking preference. In support of our hypotheses, the intervention program was feasible and acceptable. We observed a high degree of compliance with the EMI, which results in a significant decrease in sedentary time and an increase in physical activity in the EMI group, compared to the physical activity guidelines group.

22-ii) Highlight unanswered new questions, suggest future research

Highlight unanswered new questions, suggest future research.

	1	2	3	4	5	
subitem not at all important	○	○	◉	○	○	essential

Clear selection

Does your paper address subitem 22-ii?

Copy and paste relevant sections from the manuscript (include quotes in quotation marks "like this" to indicate direct quotes from your manuscript), or elaborate on this item by providing additional information not in the ms, or briefly explain why the item is not applicable/relevant for your study

This, in addition to the disproportionate risk for ADRD among this group, presents the need for culturally tailored interventions to promote health and reduce the risk for cognitive decline. This needs to be assessed in a larger sample. Importantly, any physical activity intervention needs to be sustainable in the long-term and in the community. Although we did not assess sustainability in this study it is plausible that an EMI program has the potential to translate into long-term behavior change using the principles of SEM.The sample consisted of only mid-life and older Latinos from Chicago. Due to the heterogeneity and intricacies of Latinos in the US, it is possible that Latinos in other parts of the USA differ from Latinos in Chicago. A multi-site comparison effectiveness trial should be conducted. The intervention period was short due to the proof-of-concept nature of the study, but we used alternative forms of cognitive function assessment when available to control for possible learning effects. A larger and longer duration study is needed, including a sustainability period. In addition, restrictions in place due to the COVID-19 pandemic prevented us from performing additional procedures that could have further validated our findings. For example, we were limited to performing cognitive testing from the NIH toolbox that was approved for virtual administration only. However, this study would have benefitted from administering motor function testing and Magnetic Resonance Imaging (MRI) of the brain at baseline and follow up to fully evaluate changes to functional connectivity networks. In the future, our team plans to improve representation of the study sample by expanding to include more males. We will also seek to conduct more detailed connectivity analyses, examine duration of treatment effects over time, and examine mediators and moderators of mechanisms of action.

20) Trial limitations, addressing sources of potential bias, imprecision, and, if relevant, multiplicity of analyses

20-i) Typical limitations in ehealth trials

Typical limitations in ehealth trials: Participants in ehealth trials are rarely blinded. Ehealth trials often look at a multiplicity of outcomes, increasing risk for a Type I error. Discuss biases due to non-use of the intervention/usability issues, biases through informed consent procedures, unexpected events.

	1	2	3	4	5	
subitem not at all important	○	○	◉	○	○	essential

Clear selection

Does your paper address subitem 20-i? *

Copy and paste relevant sections from the manuscript (include quotes in quotation marks "like this" to indicate direct quotes from your manuscript), or elaborate on this item by providing additional information not in the ms, or briefly explain why the item is not applicable/relevant for your study

Though several promising results have been produced, this study is not without its' limitations. The small sample size and ratio of male to female participants reduced the generalizability of our findings. The sample consisted of only mid-life and older Latinos from Chicago. Due to the heterogeneity and intricacies of Latinos in the US, it is possible that Latinos in other parts of the USA differ from Latinos in Chicago. A multi-site comparison effectiveness trial should be conducted. The intervention period was short due to the proof-of-concept nature of the study, but we used alternative forms of cognitive function assessment when available to control for possible learning effects. A larger and longer duration study is needed, including a sustainability period. In addition, restrictions in place due to the COVID-19 pandemic prevented us from performing additional procedures that could have further validated our findings. For example, we were limited to performing cognitive testing from the NIH toolbox that was approved for virtual administration only. However, this study would have benefitted from administering motor function testing and Magnetic Resonance Imaging (MRI) of the brain at baseline and follow up to fully evaluate changes to functional connectivity networks. In the future, our team plans to improve representation of the study sample by expanding to include more males. We will also seek to conduct more detailed connectivity analyses, examine duration of treatment effects over time, and examine mediators and moderators of mechanisms of action.

21) Generalisability (external validity, applicability) of the trial findings

NPT: External validity of the trial findings according to the intervention, comparators, patients, and care providers or centers involved in the trial

21-i) Generalizability to other populations

Generalizability to other populations: In particular, discuss generalizability to a general Internet population, outside of a RCT setting, and general patient population, including applicability of the study results for other organizations

	1	2	3	4	5	
subitem not at all important	○	○	◉	○	○	essential

Clear selection

Does your paper address subitem 21-i?

Copy and paste relevant sections from the manuscript (include quotes in quotation marks "like this" to indicate direct quotes from your manuscript), or elaborate on this item by providing additional information not in the ms, or briefly explain why the item is not applicable/relevant for your study

Our sample is highly representative of the Chicago Latino population and the results are generalizable. 16 The intervention was culturally and individually tailored and delivered in real-time to participants.

21-ii) Discuss if there were elements in the RCT that would be different in a routine application setting

Discuss if there were elements in the RCT that would be different in a routine application setting (e.g., prompts/reminders, more human involvement, training sessions or other co-interventions) and what impact the omission of these elements could have on use, adoption, or outcomes if the intervention is applied outside of a RCT setting.

	1	2	3	4	5	
subitem not at all important	○	○	●	○	○	essential

Clear selection

Does your paper address subitem 21-ii?

Copy and paste relevant sections from the manuscript (include quotes in quotation marks "like this" to indicate direct quotes from your manuscript), or elaborate on this item by providing additional information not in the ms, or briefly explain why the item is not applicable/relevant for your study

The use of the Fitbit activity tracker that is widely available means that the intervention is easily scalable.

OTHER INFORMATION

23) Registration number and name of trial registry

Does your paper address CONSORT subitem 23? *

Copy and paste relevant sections from the manuscript (include quotes in quotation marks "like this" to indicate direct quotes from your manuscript), or elaborate on this item by providing additional information not in the ms, or briefly explain why the item is not applicable/relevant for your study

(ClinicalTrials.gov NCT04507464; https://www.clinicaltrials.gov/study/NCT04507464)

24) Where the full trial protocol can be accessed, if available

Does your paper address CONSORT subitem 24? *

Cite a Multimedia Appendix, other reference, or copy and paste relevant sections from the manuscript (include quotes in quotation marks "like this" to indicate direct quotes from your manuscript), or elaborate on this item by providing additional information not in the ms, or briefly explain why the item is not applicable/relevant for your study

(ClinicalTrials.gov NCT04507464; https://www.clinicaltrials.gov/study/NCT04507464)

25) Sources of funding and other support (such as supply of drugs), role of funders

Does your paper address CONSORT subitem 25? *

Copy and paste relevant sections from the manuscript (include quotes in quotation marks "like this" to indicate direct quotes from your manuscript), or elaborate on this item by providing additional information not in the ms, or briefly explain why the item is not applicable/relevant for your study

This work was supported by the National Institute on Aging of the National Institutes of Health grants P30AG022849 (UGB) and R24AG064191.

X27) Conflicts of Interest (not a CONSORT item)

X27-i) State the relation of the study team towards the system being evaluated

In addition to the usual declaration of interests (financial or otherwise), also state the relation of the study team towards the system being evaluated, i.e., state if the authors/evaluators are distinct from or identical with the developers/sponsors of the intervention.

	1	2	3	4	5	
subitem not at all important	○	○	◉	○	○	essential

Clear selection

Does your paper address subitem X27-i?

Copy and paste relevant sections from the manuscript (include quotes in quotation marks "like this" to indicate direct quotes from your manuscript), or elaborate on this item by providing additional information not in the ms, or briefly explain why the item is not applicable/relevant for your study

Conflicts of Interest: None to report

About the CONSORT EHEALTH checklist

As a result of using this checklist, did you make changes in your manuscript? *

- ○ yes, major changes
- ◉ yes, minor changes
- ○ no

What were the most important changes you made as a result of using this checklist?

Your answer

How much time did you spend on going through the checklist INCLUDING making changes in your manuscript *

At least 3 hours. This is excessive. A simple checklist should suffice.

As a result of using this checklist, do you think your manuscript has improved? *

- ○ yes
- ◉ no
- ○ Other:

Would you like to become involved in the CONSORT EHEALTH group?

This would involve for example becoming involved in participating in a workshop and writing an "Explanation and Elaboration" document

- ○ yes
- ◉ no
- ○ Other:

Clear selection

Any other comments or questions on CONSORT EHEALTH

Your answer

STOP - Save this form as PDF before you click submit

To generate a record that you filled in this form, we recommend to generate a PDF of this page (on a Mac, simply select "print" and then select "print as PDF") before you submit it.

When you submit your (revised) paper to JMIR, please upload the PDF as supplementary file.

Don't worry if some text in the textboxes is cut off, as we still have the complete information in our database. Thank you!

Final step: Click submit !

Click submit so we have your answers in our database!

Submit Clear form

Never submit passwords through Google Forms.

This content is neither created nor endorsed by Google. Report Abuse - Terms of Service - Privacy Policy

Google Forms
